# Maternal diet alters human milk oligosaccharide composition with implications for the milk metagenome

**DOI:** 10.1038/s41598-020-79022-6

**Published:** 2020-12-16

**Authors:** Maxim D. Seferovic, Mahmoud Mohammad, Ryan M. Pace, Melinda Engevik, James Versalovic, Lars Bode, Morey Haymond, Kjersti M. Aagaard

**Affiliations:** 1 Division of Maternal-Fetal Medicine, Department of Obstetrics and Gynecology, Baylor College of Medicine and Texas Children’s Hospital, One Baylor Plaza, Jones 314, Houston, TX 77030 USA; 2 Department of Pediatrics, Children’s Nutrition Research Center, Baylor College of Medicine, Houston, TX 77030 USA; 3 Department of Pathology and Immunology, Baylor College of Medicine and Texas Children’s Hospital, Houston, TX 77030 USA; 4 Division of Neonatology and Division of Gastroenterology, Hepatology and Nutrition, Department of Pediatrics, Larsson-Rosenquist Foundation Mother-Milk-Infant Center of Research Excellence, University of California San Diego, La Jolla, CA 92093 USA; 5 Department of Molecular and Human Genetics, Baylor College of Medicine, Houston, TX 77030 USA; 6 Department of Molecular and Cell Biology, Baylor College of Medicine, Houston, TX 77030 USA; 7 Food Science and Nutrition Department, National Research Centre, El Buhouth St., Dokki, Cairo, Egypt

**Keywords:** Developmental biology, Molecular medicine

## Abstract

Human milk is the optimal nutrition source for infants, and oligosaccharides represent the third most abundant component in milk after lactose and fat. Human milk oligosaccharides (HMO) are favorable macromolecules which are, interestingly, indigestible by the infant but serve as substrates for bacteria. Hypothesizing that the maternal diet itself might influence HMO composition, we sought to directly determine the effect maternal diet on HMO and the milk bacteria. Employing a human cross-over study design, we demonstrate that distinct maternal dietary carbohydrate and energy sources preferentially alter milk concentrations of HMO, including fucosylated species. We find significant associations between the concentration of HMO-bound fucose and the abundance of fucosidase (a bacterial gene that digests fucose moieties) harbored by milk bacteria. These studies reveal a successive mechanism by which the maternal diet during lactation alters milk HMO composition, which in turn shapes the functional milk microbiome prior to infant ingestion.

## Introduction

Human milk is recommended as the optimal nutrition source for neonates and infants as it confers protection against both immediately life-threatening conditions such as necrotizing enterocolitis, as well as later in life chronic diseases such as obesity, diabetes and inflammatory bowel disease^1–4^. However, cross-fostering studies in mice have shown that exposure to a high fat maternal diet during lactation is sufficient to mitigate these beneficial effects and alternately render metabolic and gastrointestinal dysfunction in the nursing offspring^5–7^. This suggests that (at least in mice) the maternal lactational diet in and of itself is capable of modulating the beneficial composition of milk. However, the mechanisms by which such common maternal dietary modifications might alter the metabolism of her offspring via the breastmilk have yet to be elucidated.

One such candidate mechanistic link is the milk microbiome, given its presumptive role in contributing (at least in part) to the colonization and functional shaping of the developing infant microbiome^8–18^. Like breast feeding, alterations to the early infant microbiome have demonstrated benefits, like decreased necrotizing enterocolitis, and later in life chronic diseases, and immune function^19–21^. Human milk contains a low abundance, low biomass, and yet surprisingly diverse community of bacteria that has been suggested to augment early and initial seeding of the infant’s gut via breastfeeding (e.g., *Staphylococcus*, *Streptococcus*, *Lactobacillus*)^22^. Multiple studies have demonstrated that the infant stool microbiome of breastfed newborns, significantly varies from that formula fed, driven by the diverse microbial nutrients in human milk^8–10^. Data from our laboratory and others suggest that a high fat maternal diet during gestation and lactation causes near and long-term dysbiosis in the offspring gut microbiome, even after weaning onto a control diet^9–15^. Although challenging to distinguish between the effect of the maternal diet during gestation versus lactation, these and other studies^16^ suggest that the maternal lactation diet alters components of milk that play a role in structuring the offspring gut microbiome, thereby potentially influencing lifelong metabolic health.

While our previous work explored the effect of maternal diet on the macronutrient and micronutrient composition of human milk^23^, very few studies have explored the impact of maternal diet on its bioactive components. Human milk oligosaccharides (HMO) and the milk microbiome are particularly interesting candidates, as both are known to be associated with alterations of the neonatal and infant gut microbiome^24,25^. HMO are a diverse group of complex glycans composed of five main monosaccharides whose complexity is rendered by the diversity of their glycosidic bonds. Despite this diversity and complexity, a relatively small number of oligosaccharide structures dominate in human milk^26^, all of which are largely indigestible by the infant. HMO are presumed to play a beneficial role in infant health by favoring proliferation of beneficial bacteria, acting as “decoys” for pathogenic microbes, providing substrates essential for infant development, and establishing and maintaining the gut epithelial barrier^24–31^. For example, Charbonneau et al.previously demonstrated in a human cohort of Malawian mothers with undernourished and growth-stunted infants that there was decreased total, sialylated, and fucosylated HMO in their milk samples^29^. In a series of elegant experiments, these investigators were able to show that cultivated bacterial strains from the stool of a 6 month old growth-stunted Malawian infant could colonize gnotobiotic mice. When these colonized mice were supplemented with sialylated HMO, there was both restoration of the microbiome composition and accompanying growth of the mice in an insulin-independent manner^29^. These findings are consistent with those of others documenting an association between HMO composition in the infant stool and the microbiome, as well as varying milk oligosaccharide concentrations and profiles by virtue of maternal geography as a likely reflection of maternal genetic and environmental, fixed and modifiable factors^24,30–38^. However, to date, no study has specifically demonstrated a causal relationship between the maternal diet, the milk HMO profile, nor the functional microbiome of breast milk. In the current study we sought to determine if specifically altering crucial substrates in the maternal diet during lactation would function to drive the composition of specific HMO, thereby shaping both the community and functional profile of the milk microbiome.

## Results

### Cross-over study design

**Figure 1:**
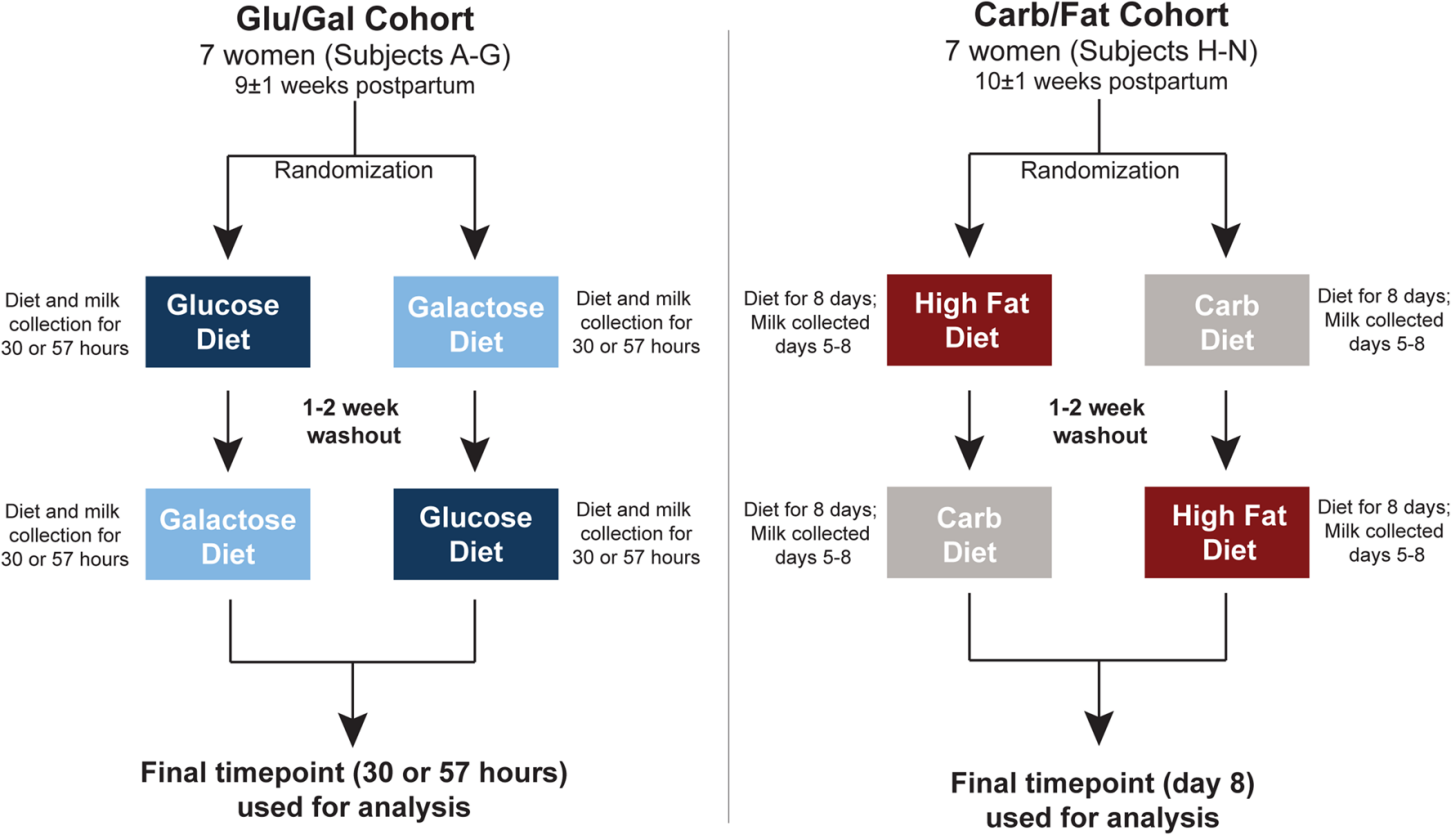
Study design: single-blinded cross-over dietary intervention studies. Two separate single-blinded cross-over dietary intervention studies were performed to evaluate the impact of maternal diet on human milk. In the first study (Glu/Gal Cohort, left), lactating women 8–11 weeks postpartum were randomly assigned to receive a liquid, isocaloric, isonitrogenous drink containing either glucose or galactose as the sole carbohydrate source for a total of 30–57 h. The sugar treatment provided 60% of their daily estimated energy requirement, and a standardized mix of protein and essential fatty acids were provided to supplement the treatment. Milk samples were collected as specified, and following a 1–2 week washout period each woman received the alternative treatment (random initial allocation); thus paired milk samples were collected from each subject, allowing each subject to serve as her own control. In the second study (Carb/Fat Cohort, right), a second cohort of 7 different lactating women 9–12 weeks postpartum were randomly assigned to receive either a high fat (55% fat, 30% carbohydrate, 15% protein) or carbohydrate (25% fat, 60% carbohydrate, 15% protein) diet for 8 days consisting of packed meals with isocaloric, isonitrogenous nutrient compositions. The milk samples collected at the completion of each dietary treatment were used for analysis. Postpartum age ranges indicate mean ± standard deviation.

Using a cross-over design whereby each subject serves as her own control, samples from two previously described cohorts of healthy lactating women were utilized for this study (Fig. 1, Supplementary Table S1) ^23,32,33^. After obtaining IRB approval for each dietary intervention study, a cohort of 7 lactating women 8–11 weeks postpartum (referred hereafter to as the Glu/Gal Cohort) were randomized to first glucose or galactose as their sole carbohydrate source, and a separate cohort of 7 lactating women 9–12 weeks postpartum (referred hereafter to as the Carb/Fat Cohort) were randomized to either first carbohydrate or high fat diet as their energy source (Fig. 1). By design, these cohorts differed by lean and obese status, and comparisons are thus limited to individual subjects as a pair-wise analysis^23,32,33^. To provide rigorous control of the dietary intervention, post-partum subjects were admitted to the General Clinical Research Center (GCRC) at Baylor College of Medicine for exclusive dietary feeding and milk sample collection as described in detail in Methods and in our prior publications^23,32,33^. During their inpatient studies, subjects were strictly fed study meals and snacks over 30–57 h for the Glu/Gal Cohort and 8 days for the Carb/Fat Cohort. Following the dietary feeding and sample collection period, subjects were discharged home for a 1–2 week “wash out” interval in which they were encouraged to return to their routine diet. Consistent with a cross-over design whereby each subject serves as her own control, subjects returned and were given the second galactose or glucose feeding, or high fat or carbohydrate feeding (Fig. 1). Milk samples collected at the bedside in the GCRC following completion of each dietary treatment were used for HMO and milk microbiome analysis. Maternal age, postnatal age, race, and ethnicity did not significantly differ between the two cohorts, while (by design) maternal body mass index (BMI) was significantly higher in the Glu/Gal Cohort (BMI: 34.5 ± 4.6) compared to the Carb/Fat Cohort (BMI 23.2 ± 1.7) (Supplementary Table S1). A mother’s secretor status reflects her blood group antigens, and determines her ability to synthesize certain HMO and therefore greatly influences her HMO profile; thus the secretor status for each subject was evaluated as described in “Methods”^34^ and was not statistically different between cohorts (Supplementary Table S1).

### Maternal diet predictably and significantly impacts specific HMO concentrations in human milk

**Figure 2:**
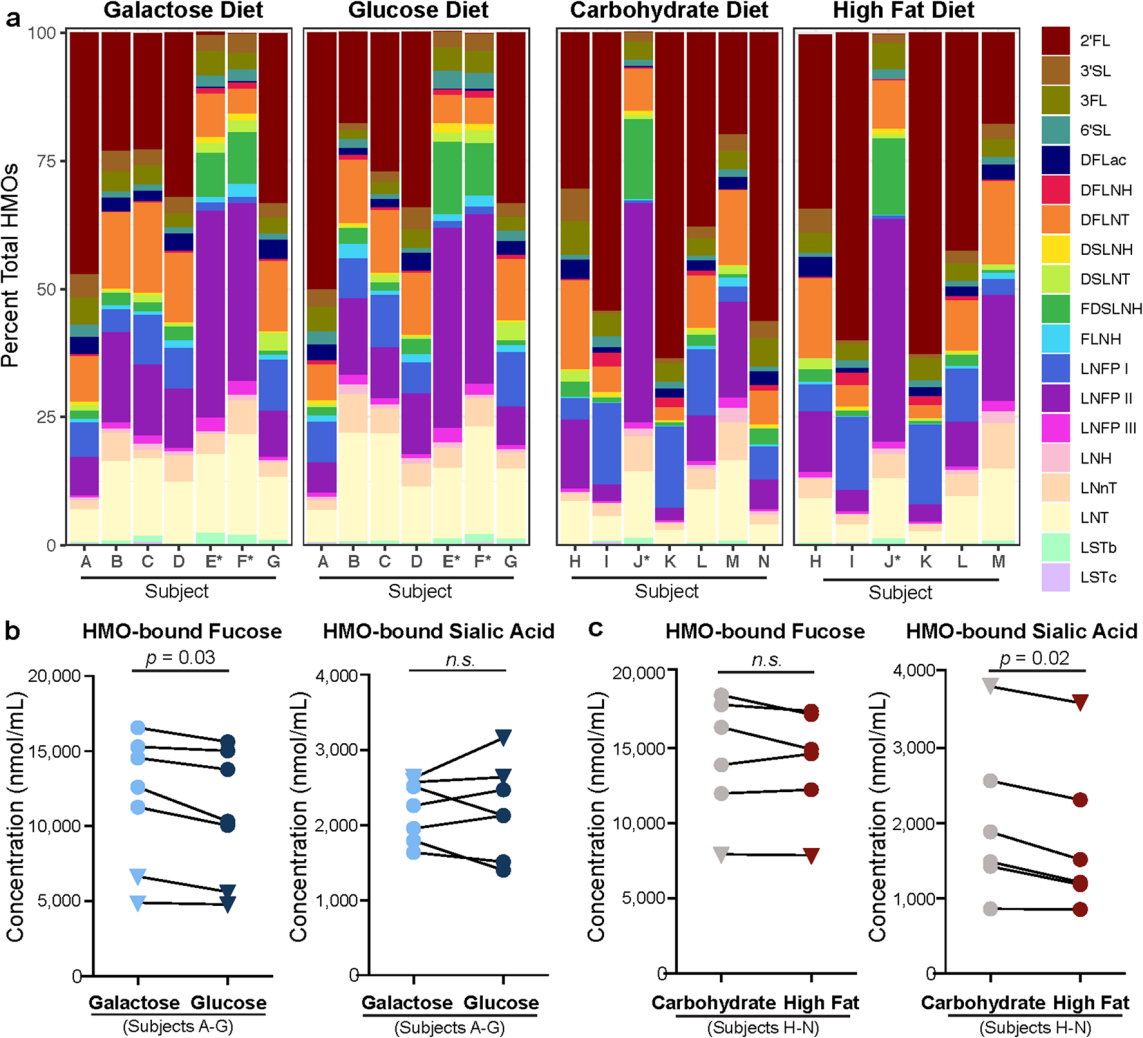
Maternal diet alters the HMO profile of human milk. (**a)** Relative abundance of individual HMO in each cohort based on molar concentration (green: fucosylated HMO, purple: sialylated HMO; A–N: subject identifiers; asterisks: non-secretors). (**b)** HMO-bound fucose is significantly decreased after glucose diet relative to galactose diet (paired t-test, *p* = 0.03), with no discernable change in HMO-bound sialic acid. (**c)** High fat diet significantly decreases HMO-bound sialic acid (paired t-test, *p* = 0.02), with no discernable change in HMO-bound fucose. Circles: secretors; triangles: non-secretors. *2′FL* 2′-fucosyllactose, *3FL* 3-fucosyllactose, *DFLac* difucosyllactose, *LNFP* lacto-*N*-fucopentaose, *DFLNT* difucosyllacto-*N*-tetraose, *FLNH* fucosyllacto-*N*-hexaose, *DFLNH* difucosyllacto-*N*-hexaose, *3′SL* 3′-sialyllactose, *6′SL* 6′-sialyllactose, *LST* sialyllacto-*N*-tetraose, *DSLNT* disialyllacto-*N*-tetraose, *DSLNH* disialyllacto-*N*-hexaose, *FDSLNH* fucosyl-disialyllacto-*N*-hexaose, *LNnT* lacto-*N*-neotetraose, *LNT* lacto-*N*-tetraose, *LNH* lacto-*N*-hexaose. In the high fat dietary intervention, 1 subject’s sample (“subject N”) had an insufficient volume (< 20 μl) to rerun a when QC for a high quality chromatogram failed^29^. As a result, subject N was dropped from the paired analysis shown in panel (**c**).

HMO concentrations in paired milk samples from each subject in both the Glu/Gal Cohort and Carb/Fat Cohort were quantified using High-Performance Liquid Chromatography (HPLC) (Fig. 2a, Supplementary Table S2). In the high fat dietary intervention, 1 subject’s sample (“subject N”) had an insufficient volume (< 20 μl) to rerun a when quality control (QC) for a high quality chromatogram failed^35^. Milk samples from both cohorts were comprised of a diversity of HMO species with over 70% being fucosylated or sialylated, and notable reductions of 2′-fucosyllactose (2′FL) and lacto-*N*-fucopentaose (LNFP) I in non-secretor subjects, consistent with their inability to synthesize α1-2-fucosylated HMO due to the inactive FUT2 enzyme^34^. HMO profiles were interrogated at the level of individual HMO as well as total molar concentration of HMO-bound fucose and HMO-bound sialic acid, which is the molar amount of fucose and sialic acid residues bound to all fucosylated and all sialylated HMO, respectively (raw data in supplementary Table S2). Both dietary interventions resulted in a significant change in the HMO profile across paired samples. In the Glu/Gal Cohort, the concentration of total HMO-bound fucose was reduced in the glucose diet relative to the galactose diet (*p* = 0.03, Fig. 2b), while no single fucosylated HMO was statistically different between diets (Supplementary Figure S1); this is as anticipated due to the high numbers of individual HMO species and the relatively small cohort size^34,35^. In general, individual HMO increased as much as 50% with galactose and 30% with high fat diet (FLNH), while other HMO were remarkably stable. In the Carb/Fat Cohort, the concentration of total HMO-bound sialic acid was reduced in the high fat diet relative to the carbohydrate diet (Fig. 2c) (*p* = 0.02), though no individual sialylated HMO were statistically different between diets following multiple comparisons correction with FDR < 0.05 in the 6 subjects paired samples available for analysis (Supplementary Figure S1).

### Maternal diet is associated with an altered functional metagenomic profile of the milk microbiome

**Figure 3:**
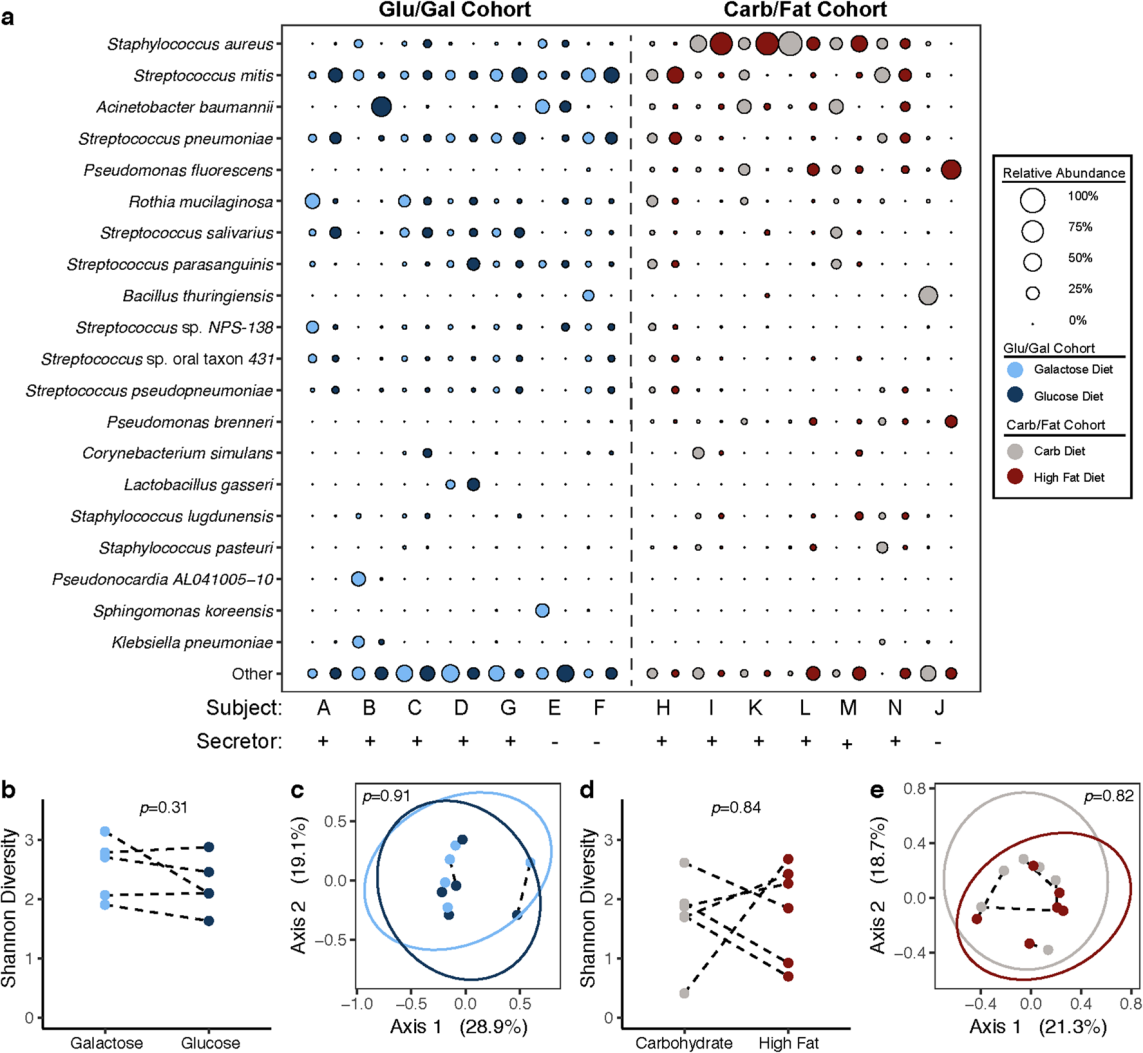
Species-level profile of milk microbiome by WGS sequencing reveals overall minimal discernable impact of maternal diet on taxonomic composition. (**a)** WGS sequencing of the milk microbiome reveals relative abundance of most abundant bacterial species. No significant difference in alpha diversity by diet is detected in Glu/Gal Cohort (**b**, *p* = 0.31, Mann–Whitney) nor Carb/Fat Cohort (**d**, *p* = 0.84, Mann–Whitney). Principal coordinate analysis (PCoA, Binary Jaccard) of milk microbiome bacterial species reveals no significant clustering by diet in the Glu/Gal Cohort (**c**, *p* = 0.91, PERMANOVA) nor Carb/Fat cohort (**e**, *p* = 0.82, PERMANOVA). For panels (**b**)–(**e**), lines connecting points indicate paired dietary samples from the same subject. Ellipses indicate 95% confidence interval of group centroids.

Given our observations regarding the impact of maternal diet on HMO composition, we next sought to determine if and how maternal diet affects the milk microbiome. Whole genome shotgun (WGS) sequencing was used to generate in-depth and refined profiles of the taxonomic and functional gene composition of the human milk microbiome, alongside “kit negative” extraction specimens controls for identification of potential contaminant taxa analyzed on decontamination pipelines (Supplementary Figures S2–S6). As shown in Supplementary Tables S3a and S3b, there was no significant variation in any subject’s metagenomics sequencing depth nor Good’s Coverage by virtue of diet alone. Importantly, and consistent with previous studies^36,37^, the milk bacterial community of both cohorts was dominated by *Staphylococcus* and *Streptococcus* spp., including *Staphylococcus aureus* and *Streptococcus mitis* (Fig. 3a, Supplementary Table S3a & S3b). *Bifidobacterium* and *Lactobacillus* spp. were present but at lower abundance (Fig. 3a, Supplementary Table S3a & S3b), consistent with previous studies of the milk microbiome utilizing Next-Generation Sequencing methodologies^36^.

As expected based on the observed HMO changes, non-secretors from both cohorts were noticeably distinct from secretors. There was dominance of otherwise rare bacterial taxa such as *Bacillus* and *Pseudomonas* spp. (Fig. 3a), and pairwise comparisons of the dissimilarity of the milk microbiome of subjects from both cohorts show that the milk microbiome of secretor subjects is less similar to non-secretor subjects when compared to other secretor subjects (Kruskal–Wallis, *p* = 4.6e−8; Supplementary Figure S2). Given that secretor status in both cohorts demonstrated significant association with microbiome composition, and has been associated with human milk microbiome changes elsewhere^37,38^, we removed non-secretors rom all subsequent metagenomic analysis so as not to confound the effect of diet. This included two participants from the Glu/Gal cohort and one from the Carb/Fat cohort (Supplementary Table S1).

**Figure 4:**
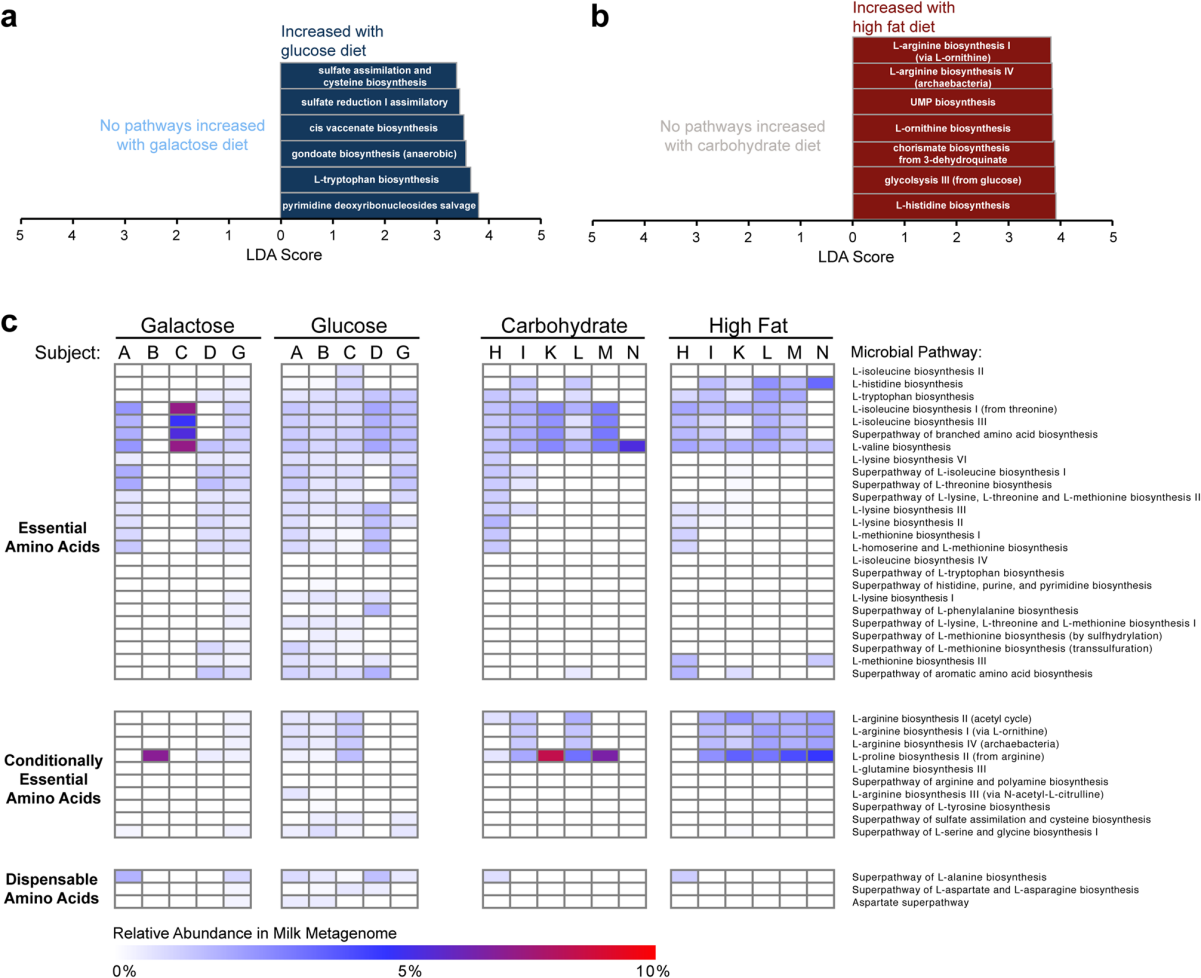
Despite overall lack of impact on the taxonomic community composition, maternal diet significantly alters the metagenomic composition of the milk microbiome. Linear Discriminant Analysis (LDA) Effect Size (LEfSe) of the milk microbial metagenome profiled by WGS sequencing identifies differentially abundant metabolic pathways (LDA score > 2.0, *p* < 0.05) in the glucose versus galactose diet (**a**) and carbohydrate versus high fat diet (**b**). (**c)** Heat map of amino acid biosynthetic pathways reveals carriage of essential and conditionally essential amino acid biosynthetic pathways by milk bacteria.

After applying sequencing and analytic methods controlling for possible contamination, the taxonomic and metagenomic composition of the milk microbiome was interrogated using WGS sequencing (i.e., shotgun whole metagenomic sequencing) to optimally quantify the abundance of bacterial species and determine the presence of genetically-encoded bacterial metabolic pathways. We found that the taxonomic composition of the milk microbiome was minimally affected by maternal diet (Fig. 3). Neither alpha diversity (Shannon diversity, Fig. 3b, d) nor beta diversity (Bray Curtis dissimilarity, Fig. 3c, e) varied significantly by maternal diet in either cohort. However, while taxonomic composition did not vary by diet^39^, feature selection by Linear Discriminant Analysis (LDA) Effect Size (LEfSe)^40^ revealed that the abundance of multiple metabolic pathways varied by maternal diet in both the Glu/Gal Cohort and Carb/Fat Cohort (Fig. 4a, b). Many of the pathways affected by the maternal diet are involved in amino acid biosynthesis including the biosynthesis of the essential amino acids tryptophan and histidine. Interestingly, further evaluation of the abundance of all amino acid biosynthetic pathways demonstrates that the milk microbiome possesses the metagenomic capacity to synthesize numerous essential and conditionally essential amino acids (Fig. 4c).

### Maternal diet-driven HMO concentrations are significantly associated with milk microbiome composition

**Figure 5:**
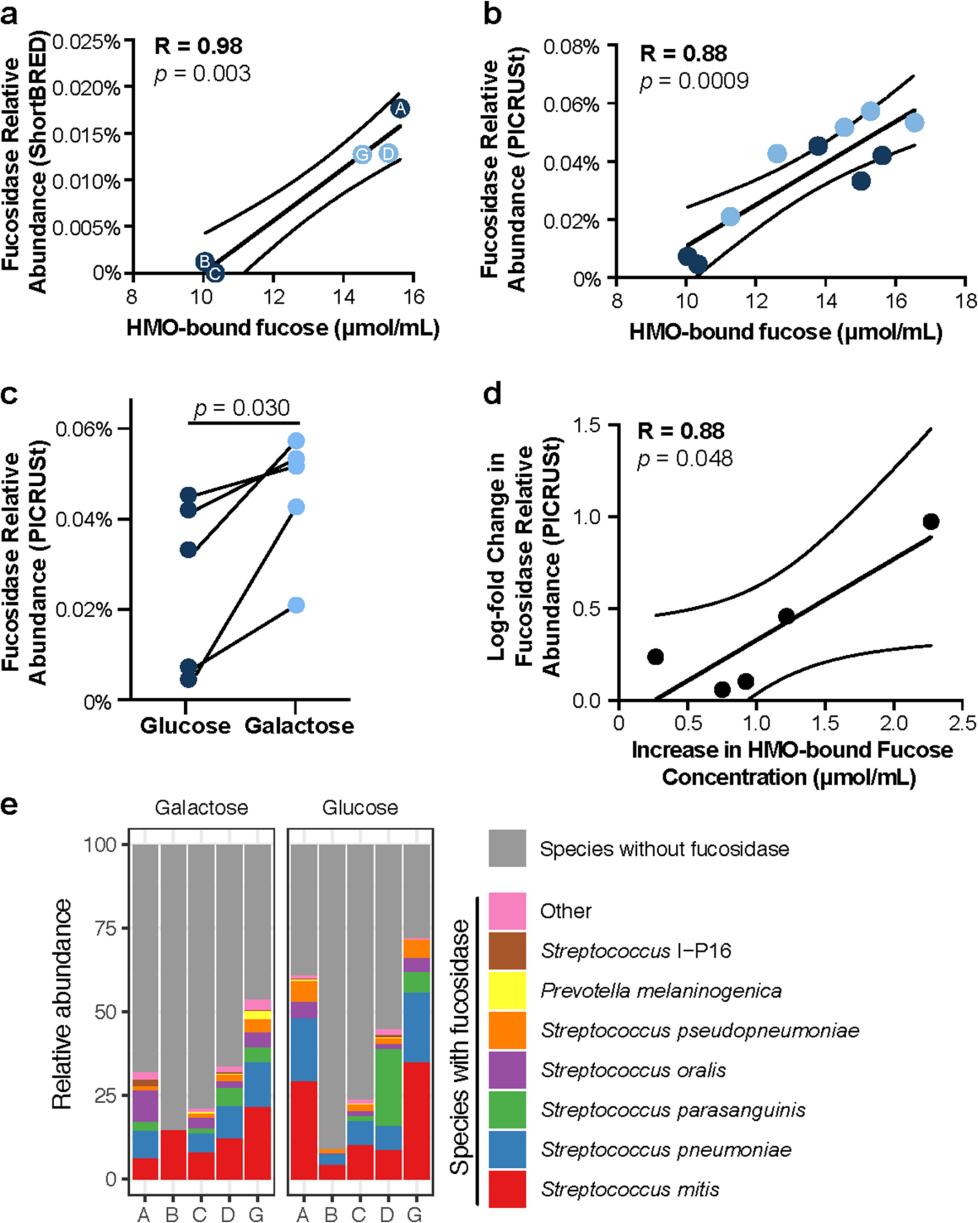
Levels of HMO-bound fucose are specifically and significantly associated with bacterial fucosidase abundance in the milk microbiome. (**a)** Linear regression reveals tight association between HMO-bound fucose concentration and bacterial fucosidase (K01206) abundance as determined by ShortBRED in Glu/Gal Cohort (R = 0.98, *p* = 0.003, linear regression). As not all samples had sufficient sequence coverage for ShortBRED analysis, samples included are indicated by letters (subject ID) and colors (light blue: galactose diet, dark blue: glucose diet). (**b)** Linear regression of HMO-bound fucose and bacterial fucosidase abundance determined by PICRUSt for all samples in Glu/Gal Cohort (R = 0.88, *p* = 0.0009, linear regression) confirms tight association between levels of HMO-bound fucose and fucosidase abundance. (**c)** Bacterial fucosidase abundance (PICRUSt) increases with galactose relative to glucose diet (*p* = 0.030, paired t-test), which is concordant with the increase in HMO-bound fucose with galactose relative to glucose diet (Fig. 2). (**d)** The magnitude of the increase in bacterial fucosidase abundance (PICRUSt) with diet is proportionate to the increase in HMO-bound fucose (R = 0.88, *p* = 0.048, linear regression), suggesting that the increase in HMO-bound fucose may drive a proportionate increase in bacteria with fucosidase genes. (**e)** Relative abundance of likely non-contaminant bacterial species with fucosidase (as annotated in UniProt) in each sample of the Glu/Gal Cohort as determined by WGS sequencing reveals the majority of fucosidase-possessing taxa are *Streptococcus* spp. Error bars in linear regression analyses indicate 95% confidence intervals.

We next sought to evaluate the interaction between HMO composition and the milk microbiome. We first semi-quantified the abundance of bacterial fucosidase in the milk microbiome, the microbial genes that enable removal of fucose residues from sugars^41^. We found in the Glu/Gal Cohort, the concentration of HMO-bound fucose is significantly associated with the relative abundance of bacterial fucosidase (Fig. 5). We first quantified fucosidase in the WGS metagenomic data set using ShortBRED^42^. A list of orthologues was obtained from Kyoto Encyclopedia of Genes and Genomes (KEGG) Orthology (KO) group K01206). KEGG may be freely used by academic users (www.genome.jp/kegg/legal.html). ShortBred then created the gene markers from annotated fucosidase genes and quantified these gene markers in the WGS metagenomic data. Given that high sequencing coverage (greater than 15,000 annotated WGS reads) is necessary to accurately quantify individual KOs in metagenomic data sets^43^, we only included samples with greater than 15,000 mapped bacterial WGS reads in our analysis (N = 16, Figs. 4 and 5a, Supplementary Table S4 and S5). To cross-validate these results across varying analytic and sequencing methodologies, we also quantified fucosidase relative abundance in all samples using inferred metagenomics, which more accurately quantifies abundance of individual KOs in samples with lower sequencing coverage^43^ (Fig. 5b). We found that just as HMO-bound fucose concentrations are increased with the galactose diet, the inferred abundance of fucosidase is also increased with the galactose diet (Fig. 5c). Furthermore, the magnitude of this increase in fucosidase is proportionate to the magnitude of the increase in HMO-bound fucose across paired samples (Fig. 5d), suggesting that the diet-induced increase in HMO-bound fucose may be driving the observed change in fucosidase abundance.

We next determined which bacterial species contribute to the presence of fucosidase in these samples. Using WGS sequencing to identify and quantify individual species in the Glu/Gal Cohort we queried the UniProt protein database^44^ to determine which of the likely non-contaminant species (Supplementary Figure S5) possess an annotated fucosidase (K01206) gene (Fig. 5e), and discovered the majority of the species are *Streptococcus* spp., including *Streptococcus mitis, Streptococcus pneumoniae*, and *Streptococcus oralis*. This was consistent with the results of our WGS metagenomics ShortBRED analysis, which identified multiple annotated fucosidase genes in these samples (Supplementary Table S5). Notably, there was no significant difference in mapped V4 bacterial reads between samples with > 15,000 mapped bacterial WGS reads and samples with < 15,000 mapped bacterial WGS reads (Supplementary Figure S5). We achieved a high degree of concordance between ShortBRED (WGS) and PICRUSt (16S) results for samples with sufficient WGS sequencing coverage to perform ShortBRED analysis and poor concordance between samples with fewer than 15,000 mapped bacterial WGS reads (Supplementary Figure S5), consistent with the previously published finding that PICRUSt more accurately quantifies gene abundance than WGS metagenomics in samples with fewer than 15,000 annotated WGS reads^43^. We performed an additional validation by querying the UniProt database^44^ for all bacterial species identified by Kraken in the WGS data to determine which species are annotated to possess the fucosidase gene, which enabled us to determine the total relative abundance of species with fucosidase (Fig. 5, Supplementary Figure S5). We found the total relative abundance of species with annotated fucosidase to be tightly correlated to PICRUSt results for fucosidase abundance in all samples (Supplementary Figure S5).

**Figure 6:**
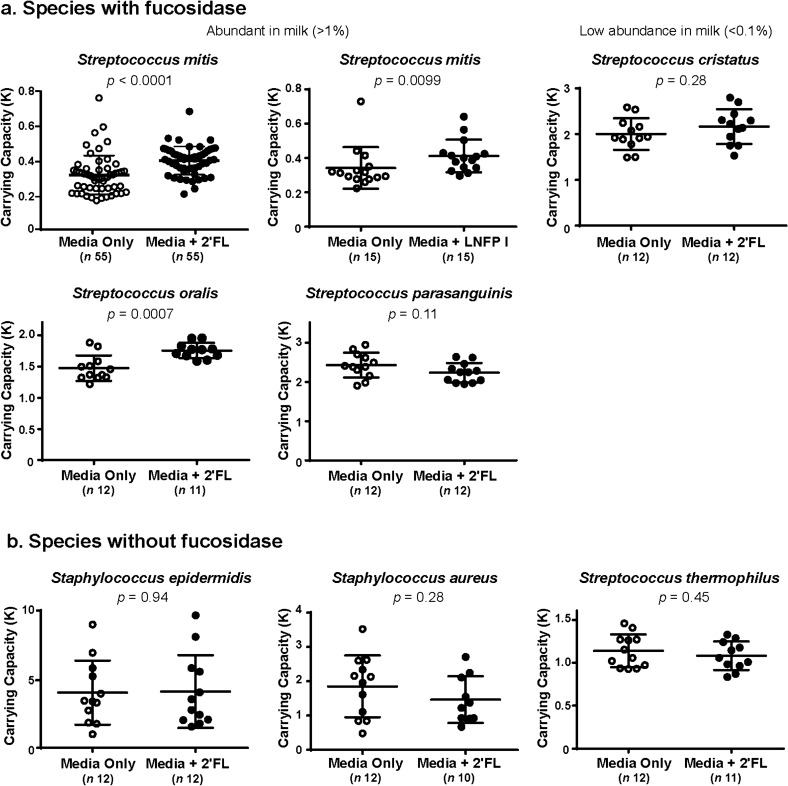
Fucosylated HMO confer significantly increased carrying capacity and growth advantage to *Streptococcus spp.* with annotated fucosidase gene found to be abundant in human milk. (**a)** Evaluation of in vitro carrying capacity (K) calculated from in vitro measures of *Streptococcus spp.* with annotated fucosidase reveals that 2′-fucosyllactose (2′FL), the most abundant fucosylated HMO in secretor subjects from the Glu/Gal Cohort, confers an increased carrying capacity to *Streptococcus mitis* and *Streptococcus oralis*, two abundant species in milk from secretors in the Glu/Gal Cohort (average relative abundance > 1%). Addition of lacto-*N*-fucopentaose (LNFP) I also confers an increased carrying capacity to *Streptococcus mitis*, the most abundant *Streptococcus* spp. in the Glu/Gal Cohort. Addition of 2′FL did not confer a discernable growth advantage (not shown) nor carrying capacity to *Streptococcus parasanguinis* nor *Streptococcus cristatus*, the latter of which is found at very low abundance in milk samples (average relative abundance < 0.1%). (**b)** Of the species without annotated fucosidase, there was no significantly increased carrying capacity with the addition of 2′FL. Error bars indicate mean ± standard deviation.

While consumption of fucosylated HMO by *Bifidobacterium* and *Lactobacillus* spp. has been extensively studied, digestion of fucosylated HMO by *Streptococcus* spp. has been largely unexplored. We selected the abundant subset of the *Streptococcus* spp. found in our metagenomic data and compared in vitro growth of these species with and without supplementation with individual fucosylated HMO found to be abundant in our samples. We discovered that 2′FL and LNFP I both confer a growth advantage to *Streptococcus mitis*, the most abundant *Streptococcus* spp. in the Glu/Gal Cohort, and 2′FL confers a growth advantage to *Streptococcus oralis* (Fig. 6a)*.* Both of these *Streptococcus* spp. possess an annotated fucosidase gene and are abundant in the milk samples from secretors in the Glu/Gal Cohort. *Streptococcus parasanguinus* and *Streptococcus cristatus* had no discernable growth advantage with 2′FL (Fig. 6a). Notably, *Streptococcus cristatus*, while annotated to possess a fucosidase gene, was found at very low abundance in our milk samples (average relative abundance < 0.1%). As expected, we found that 2′FL does not confer a growth advantage to the two most abundant *Staphylococcus* spp. in our samples as well as *Streptococcus thermophilus,* all of which do not possess annotated fucosidase genes (Fig. 6b). This demonstrates that fucosylated HMO drive proliferation of a subset abundant *Streptococcus* spp. with fucosidase found in human milk, while abundant taxa without fucosidase genes show no discernable growth advantage with fucosylated HMO by carrying capacity.

We additionally interrogated the relationship between HMO-bound sialic acid and sialidase gene abundance in the Glu/Gal Cohort and found no significant association (Supplementary Figure S3). This may indicate that fucosylated HMO are a stronger driver of milk microbiome composition than sialylated HMO due to the higher abundance of fucosylated HMO in human milk (Fig. 2). We found no significant association between HMO-bound fucose and fucosidase abundance nor HMO-bound sialic acid and sialidase abundance in the Carb/Fat Cohort; this is likely explained by the low levels of bacteria possessing fucosidase and sialidase in this cohort, as *Staphylococcus* spp. dominate in the majority of samples in this cohort, and *Streptococcus* spp. abundance is significantly lower (Supplementary Figure S3). Finally, we interrogated the absolute quantification of HMO concentrations in our subjects in relation to others reported in the literature (Supplementary Figure S8), as well as a product of weeks post-partum (Supplementary Figure S9). Our quantified concentration of HMO in milk is in alliance with other values reported in the literature (Supplementary Figure S8), and did not significantly vary by virtue of week post-partum nor randomized order of dietary feeding intervention (*e.g.,* glucose-galactose or galactose-glucose, nor carbohyrdate-high fat or high fat-carbohydrate; Supplementary Figure S9).

## Discussion

It is well-known that numerous metabolic adaptations occur during lactation that support milk synthesis without jeopardizing the maternal metabolic milieu^23–35^. Specifically, oral galactose provides a substrate for glucose conversion and lactose synthesis that results in endogenous fat mobilization and oxidation during meal absorption secondary to the lower plasma insulin concentrations^32^. Conversely, Mohammad et al. previously demonstrated that when compared with a carbohydrate rich diet, an isonitrogenous, isocaloric high fat diet increased milk fat, energy output and energy expenditure but not gluconeogenesis^23^.

The current study furthers prior findings by first testing the impact of strict dietary interventions that vary the glycemic index of the carbohydrate source (i.e., glucose of 100 versus galactose of 25) or vary the energy source (i.e., carbohydrates versus fat) on HMO composition in a cross-over cohort comprised of lactating women. By design these cohorts differed with respect to maternal lean and obese status, thus all comparisons are limited to single subject pair-wise analysis, and no conclusions are drawn between cohorts. After controlling for potential kit and environmental contamination and accounting for secretor status, we observed a significant impact of maternal dietary feeding on the overall HMO-bound sialic acid and fucose composition, which appeared to be driving the milk microbiome metagenomic functional but not taxonomic variation. To validate these observations, we then performed a series of in vitro studies with a subset of the *Streptococcus* spp. found in our metagenomic data and compared in vitro growth of these species with and without supplementation with individual fucosylated HMO found to be abundant in our samples. We discovered that fucosylated HMO drove proliferation of a subset of abundant *Streptococcus* spp. with fucosidase found in human milk, while abundant taxa without fucosidase genes failed to demonstrate a discernable growth advantage with fucosylated HMO.

There are several limitations to our study, which are important to acknowledge. First, as with all low biomass microbial communities, these experiments are inherently susceptible to contamination by kit reagents or the environment. However, in this study, we are comparing paired samples and looking for consistency of findings when comparing identically collected, sequenced, and analyzed samples between diets. When combined with our use of contamination controls and decontamination analytic pipelines and stringent analytic methodologies, our study design allowed us to discern reliable true signal over the potential background contaminant noise. Thus the controlled nature of the diets during the inpatient stay and the cross-over dietary intervention design allowed for reliable interrogation of the effect of maternal diet on HMO composition and milk bacteria; however given the intensive subject involvement and expensive resources required to perform these type of studies, our sample size was necessarily limited. Thus, we were underpowered to perform multivariate analysis to identify associations among multiple individual HMO and bacterial taxa. As a corollary, it is possible that we were underpowered to detect individual HMO changes, as a few approached significance. However, it is important to note that differences in overall HMO-bound fucose or sialic acid availability to the infant gut microbiome can have biological relevance even if we do not observe significant changes in individual HMO concentrations. Additionally, while DNA sequencing (*i.e.,* WGS metagenomics) enables us to reliably quantify the abundance of bacterial genes involved in specific metabolic functions, we are unable to determine the relative expression of these genes and corresponding protein activity from this type of data and would rather necessitate detailed transcriptome, enzymatic, and proteomics analysis; this is outside the scope of the current study. Finally, sample the size limitation may have rendered us unable to detect subtle effects of maternal diet on individual HMO and bacterial abundances, and larger studies with longer duration of diets may reveal additional effects of maternal diet that were not discernable in our cohorts.

Despite these limitations, our study retains significance and novelty of findings. Our data is grounded in the strength of our cross-over design and reinforces the notion that human milk is not a static, but rather it is a dynamic and complex nutrition source for the developing infant which changes based on an interplay between the maternal diet and the resultant HMO and bacterial composition in human milk. Our findings arise from a series of consistent observations derived from a unique cohort of post-partum lactating women who were subjected to dietary interventions in a clinical research setting for a short time span. The strength of our cohort is its rigorous cross-over study design which allows each subject to serve as her own control, and therefore enabling direct inference between the tested dietary interventions (galactose/glucose or carbohydrate/fat) and measured variations in HMO and microbial speciation as a result of the prescribed dietary change. Our resultant observations are therefore significant for several reasons.

First, we provide evidence that maternal dietary interventions over the span of a few short days during lactation are independent modifiers of HMO composition. Previously it was known that both geography and BMI are associated with the HMO profile^35^, and the role of maternal genotype in determining specific HMO concentrations is well established^26,34^. Evaluating the independent impact of maternal diet on HMO composition in population-based cohorts is challenging due to the collinearity between maternal diet, BMI, geography, and other potential confounders such as mode of delivery and antibiotic use. However, by using a cross-over study design we were able to control for each of these factors as well as maternal genotype, and thus determine that maternal diet may function as the driver of the observed HMO changes in her milk. Given the relatively short duration of these diets, this indicates that HMO are rapidly altered in response to maternal dietary changes. We speculate that, since HMO are largely fodder for microbes, this may serve as an *ex utero* means by which she may tailor the establishment of the microbial species of the infant gut which will be necessary to someday utilize the dietary substrates that her offspring will encounter. It is important to note that this may be to trim or prune the microbiome, and does not necessarily imply colonization with milk microbes per se.

Second, we demonstrate that maternal diet significantly alters the metagenomic (but not taxonomic) composition of the milk bacteria via diet-driven alterations in the HMO constituents. In particular, we show that multiple metagenomic (bacterial encoded) pathways crucial for both carbohydrate^24–32^ and amino acid biosynthesis are altered by maternal diet. While human milk is known to contain both essential and conditionally essential free amino acids^45^, the origin of these amino acids has not been well elucidated. Intriguingly, the biosynthesis of certain essential amino acids such as lysine by the human gut microbiome has been shown to contribute to host amino acid homeostasis^46,47^. While nitrogen recycling makes it difficult to experimentally determine whether amino acids are microbial derived^47^, the presence of these biosynthetic pathways in the milk microbiome led us to speculate that the milk microbiome may play a small role in providing essential amino acids for the infant by synthesizing amino acids in human milk. Such speculations ought to consider that the actual contribution by the relatively low biomass of milk bacteria themselves would be expected to be very scant (since at least 60–70% of amino acids in human milk are derived from the maternal plasma)^45^. Indeed, it has been previously shown that amino acids sources other than the plasma free amino acid pool provide a significant portion of the substrate for milk protein synthesis^48^. In this earlier study, Sunehag and Haymond^48^ infused ^13^C-labeled leucine (an essential amino acid) and measured the sources of amino acids for milk protein synthesis using stable isotope dilution methodologies. Their observations suggesting that 20–30% (range being attributed to fed versus fasting state differences, respectively) arose from degradation of albumin and other proteins within mammary epithelial cells is of potential relevance to our current findings. However, while it is interesting to speculate that the milk bacteria could potentially factor into milk biosynthesis, we again emphasize that we feel it unlikely that the milk microbiome would be a substantial contributor to either this plasma free pool of essential or conditional amino acids. However, it is possible that these milk microbes may alternately contribute to the establishment of an infant gut microbiome with the capacity to continue producing variant amounts of essential amino acids^46,47^. This remains to be determined experimentally, and is outside the scope of our current study to do so.

Third, our observed shifts in metabolic capacity are notable in light of our increasing understanding of which gut bacteria harbor specific and sometimes rare metabolic pathways that enable production of key bioactive metabolites that impact host physiology^49,50^. Most recently, Lawson et al.^24^ observed that *Bifidobacterium* strains from the same infant have “overlapping but distinct HMO abilities”, with niche metabolic functional capabilities that are complementary suggesting that at least *Bifidobacterium* spp may act within a single infant ecosystem to maximize community nutrition utilization from human milk, specifically HMO. These findings are in alignment with our own observations, demonstrating that while the species composition did not significantly vary with short duration changes in the maternal diet, the metagenomic composition did. This is further consistent with the notion of Lawson et al.that HMO metabolism permits the sharing of resources in the milk to maximize nutrient composition from the maternal diet^24^. Our findings further expand these recent observations in two key ways. First, we demonstrate that this is not limited to infant stool and the pangenome of *Bifidobacterium* spp, but extends to the milk metagenome which is dominated by *Streptococci* and other species. Second, our findings demonstrate that the HMO speciation itself was responsive to even short duration changes in the maternal diet. While human milk contains a relatively low biomass bacterial community, the stark shifts in the infant gut bacterial composition associated with breastfeeding^13,51^, as well as the presence of shared bacterial strains between milk and the infant gut^52^, potentially suggest that vertical transmission of milk bacteria may play a role in establishing the metabolic capacity of the infant gut microbiota. Thus, while this short-term study revealed relatively subtle but significant shifts in the milk bacteria’s metabolic and functional capacity via its metagenome, chronic aberrations in maternal diet throughout the duration of lactation may have a significant impact on the gene carriage patterns of the infant gut microbiota due to the continuous supply of milk bacteria. It is possible that longer term persistent changes may contribute to the transgenerational transmission of obesity. We have previously shown that these dietary interventions alter maternal plasma glucose and insulin concentrations, as well as glycerol, palmitate, free fatty acids, and triglyderides^23,32^.

In light of the novelty of our in vivo findings with regard to HMO-associated alterations in *Streptococci*, we performed additional in vitro experiments with fucosidase and non-fucosidase containing strains. Although these strains were not necessarily initially isolated from breast milk, they nonetheless show for the first time that fucosylated HMO confer a carrying capacity difference (growth advantage) when added to cultures of *Streptococcus mitis* and *Streptoccocus oralis*. These two species possess fucosidase genes and are relatively abundant in human milk. *Streptococcus* spp. have been consistently reported as a dominant genus in the milk microbiome^36^, and our data provides further new evidence that in addition to lower abundance genera such as *Lactobacillus* and *Bifidobacterium*, *Streptococcus* spp. are also capable of altering their carrying capacity and conferring a growth advantage in media supplemented with HMO.

We speculate the significance of HMO-driven proliferation of *Streptococcus* spp. may be twofold: firstly, this may be of maternal benefit and function to help prevent mastitis by allowing *Streptococcus* spp. to outcompete potential pathobionts such as *Staphylococcus aureus,* a common causative pathogen of mastitis^53^. Since mastitis is known to be associated with a loss of diversity in the milk microbiome^53^, we speculate that HMO may serve to prevent sparsity and dysbiosis by favoring proliferation of multiple *Streptococcus* spp., thus increasing the bacterial diversity of the milk microbiome to “crowd out” pathogens. Second, HMO may serve to promote growth of commensal *Streptococcus* spp. in the infant digestive tract, in particular the oral microbiome where *Streptococcus* spp. predominate and are crucial for the establishment of complex commensal communities^54,55^. We speculate the net effect of both mechanisms would be the minimization of mastitis pathobionts in both the maternal milk and the infant mouth (*e.g., Staphylococcus aureus*); this concept is consistent with the ecologic principle of colonization resistance. On a global scale as great as 20–30% of all breastfeeding women have at least one occurrence of postpartum mastitis^56,67^, and while modern medicine in the developed world generally regards mastitis as being self-limiting or readily treatable with a short course of dicloxacillin or cephalexin, community acquired methicillin-resistant *S. aureus* (CA-MRSA) breast infections have been increasing^56^. Approximately 10% of cases result in breast abscess formation, and in under resourced settings with scant availability of both antibiotics and readily accessible care, mastitis increases the risk of transmission of HIV through breastfeeding^56,58^. A promising route of future investigation will be to explore the use of maternal dietary modification and optimization for the prevention of postpartum or puerperal mastitis; such approaches could reduce the prevalence of mastitis resulting in less antibiotic use and diminished prevalence of resistant organisms, particularly CA-MRSA, alongside an anticipated risk reduction of postpartum mother-to-child transmission of HIV.

Together, our data highlights the complex and successive interaction between maternal diet, HMO, and the constituent species which comprise the milk microbiome. While the effect of maternal diet on macronutrient composition of human milk has been previously interrogated^16^, here we suggest that maternal diet influences the community function of the milk microbiota via modulations in HMO. Our findings identify a link between intake of specific maternal dietary carbohydrate (glucose or galactose) or macronutrient energy (carbohydrate versus fat) sources during lactation with the production of macromolecules which nursing infants cannot digest or metabolize (HMO), but rather which function as metabolic substrates for commensal bacteria. We find this to be a rather remarkable suggestion as to how humans have evolved and sustained production of unique oligosaccharides during a limited but crucial interval of reproduction (lactation), evidently solely for the purpose of feeding commensal microbes present in human milk and the infant digestive tract. Given that HMO require significant energy expenditure on the part of the mother, these findings lead us to speculate that the maternal diet may tailor the composition of the milk metagenome via HMO speciation not only for the high physiological value for the infant, but for her own potential benefit in the prevention of pathogen colonization. Regardless of whether or not these speculations hold true in future studies, our current findings underscore the possibility for the maternal diet to alter infant and maternal microbial ecology even after birth via the milk and its macromolecular components.

### STAR methods

#### Contact for reagent and resource sharing

Further information and requests for resources and reagents should be directed to and will be fulfilled by the Lead Contact, Kjersti Aagaard (aagaardt@bcm.edu).

#### Experimental model and subject details

All study protocols and consents were approved by Baylor College of Medicine’s Institutional Review Board for Human Subjects and the General Clinical Research Center (GCRC) Advisory Committee (Protocols H25057, H20026, and H18173). For all subjects, the details and possible consequences of participation were discussed and written informed consent was obtained before enrollment. All experiments were performed in accordance with relevant guidelines and regulations. Given the study focus on human lactation, all subjects enrolled were adult females over 18 years of age. See Table S1 for additional cohort characteristics.

## Method details

### Cohort recruitment and characteristics

#### Study design

Details of study design and sample collection was published in our first report related to the study^23^ and other following reports^32,33^. Each woman was oriented to use a standard breast pump (Playtex, Dover, DE, USA). The breast shield or breast pump cup assembly was initially sterile and additionally washed with deionized water and autoclaved before first use. Following each breast pumping, the breast shields were first washed with tap water, dried over a clean towel, and then wiped with alcohol swaps before next use. Human milk was collected simultaneously from both breasts into sterile 4 oz milk bottles (Abbott, Columbus, OH, USA (Part # 51950). Fore-, mid-, and hind-milk aliquots (2.5 mL × 3 from each breast) were pumped and transferred from the bottles into a 15 mL sterile centrifuge tube with screw cap (VWR, Cat#21008-212). The bottles were used for one time collection only and then discarded. Samples from each breast were pooled (total of 15 ml) at each time point. Ten (10) mL were used for the milk fat globule isolation.

Milk aliquots were transferred from the bottles into the conical tube using adjustable Rainin pipets provided with 1000 µl pre sterilized filter tip (Rainin, Mettler-Toledo, LLC, Columbus, OH, USA, Cat# RTL1000F). The conical tube was mixed thoroughly and aliquoted into two different tubes. First, ~ 10 m were transferred using sterile transfer pipet (VWR cat#414004-037) into a second pre-weighed conical tube and subjected for centrifugation (5000 rpm for 15 min at 4 °C) for processing of milk fat globule for RNA extraction for gene expression and miRNA analysis. About 3.0 mL of the infranate were transferred into a 3.5 mL clean storage tube (Cell Associates, Inc. Cat# 1545) and stored at − 80 °C for the measurement of lactose and protein concentration as well as hexose enrichment in the lactose moiety. Second, approximately 3.5 mL of whole milk were transferred into a clean 3.5 mL storage tube (Cell Associates, Inc. Cat# 1545) and stored at − 80 °C. These whole milk samples were analyzed for total milk lipid content, total fatty acid and glycerol concentration and glycerol isotopic enrichments, and the final sample was subsequently used for both bacterial and HMO analyses.

For each analysis, samples were thawed simultaneously on ice under standard chemical hood and clean sterile and non-sterile pipet tips were used to obtain the required sample volume for each specific analysis (Rainin, Mettler-Toledo, LLC, Columbus, OH, USA, Cat# RTL200F, RTL200). Samples were then promptly returned back to − 80 °C freezer. Samples were limited to 2 to 3 freeze–thaw cycles. For bacterial analysis, following thawing samples on ice in the chemical hood, ~ 1 mL of whole human milk sample (2nd aliquot) was transferred using Rainin pipets equipped with 1000 µl pre-sterilized filter tip (Rainin, Mettler-Toledo, LLC, Columbus, OH, USA, Cat# RTL1000F) into a sterile DNAse & RNAse free 1.5 mL microcentrifuge tubes (Fisher Scientific , Cat# 05408129). Samples were kept in a − 80 °C freezer for a few hours then transferred on ice for microbial DNA extraction in a clean, sterile hood. For HMO, 200 µL of whole milk was transferred using Rainin pipets equipped with 200 µl pre-sterilized filter tip (Rainin, Mettler-Toledo, LLC, Columbus, OH, USA, Cat# RTL200F, RTL200) into 0.5 mL microcentrifuge tubes (USA Scientific, Ocala, FL, USA, cat# 1405-0000), centrifuged at 4 °C at 10,000 rpm for 10 min. Infranate was then transferred into a clean a 0.5 mL microcentrifuge tube and kept in the freezer at − 80 °C until shipped or transferred for further analyses (HMO or bacterial, respectively) over dry ice.

### Dietary cohorts

*Glu/Gal Cohort* Details of this cohort and study design have been previously described^32^. Healthy, obese, exclusively breastfeeding women were enrolled in this study (subjects A-G). Healthy was defined by: 1. normal physical examination, and 2. normal results for liver function tests, fasting blood glucose, hemoglobin, and glycated hemoglobin concentrations. Additionally, all women had no history of gestational diabetes nor family history of diabetes. Women were not taking any medications at the time of this study except for postpartum vitamin and mineral supplements, and all women had a negative pregnancy test. All women were exclusively breastfeeding at the time of the study and were between 6 and 12 weeks postpartum. Subjects were not selected on the basis of ethnicity, and included White, Non-Hispanic (n = 2), White, Hispanic (n = 4), and African American (n = 1) women.

Each subject was studied at the GCRC on two separate occasions separated by a 1–2 week washout period; during this washout period, their home diets were not monitored. However, during the preceding 3 days before each admission to the GCRC, subjects were instructed to eat a standardized meal (~ 15% protein, 50% carbohydrate, 35% fat). Women were admitted to the GCRC and randomly assigned to consume an isocaloric drink containing either glucose or galactose (98% purity; Sigma-Aldrich, St. Louis, MO) every 3 h. The sugar in the drink provided ~ 60% of each subject’s estimated energy requirement (EER). Protein (Beneprotein; Novartis, Minneapolis, MN) and essential fatty acids (safflower oil, > 70% linoleic acid) added to the drink provided an additional 10% of the EER, for a total of 70% of the subject’s EER. In total, the macronutrient composition of the drink was 83% carbohydrate, 10% protein, and 7% fat. No additional caloric intake was provided to account for the caloric expenditure of milk production. The EER for each subject was calculated based on the following formula:

$${\text{EER}}\,\left( {\text{kcal/d}} \right) = 448{-}\left[ {7.95 \times {\text{age}}\,\left( {\text{y}} \right)} \right] + \left\{ {{\text{PA}} \times \left[ {11.4 \times {\text{weight}}\,\left( {{\text{kg}}} \right) + 619 \times {\text{height}}\,\left( {\text{m}} \right)} \right]} \right\}$$

where PA (physical activity coefficient) = 1.16 (low activity).

Five of the subjects completed the long protocol (total of 57 h) in which they received the sugar drink every 3 h for 38 h, followed by a 13 h overnight fast, and then another 6 h consuming the sugar drink (25 mL every 15 min). During the last 13 h of the study, isotopic infusions of glucose, glycerol, and palmitate bound to human albumin were performed for kinetics studies previously described^32^. Due to time and budget restraints at the GCRC, the remaining two subjects completed a shorter protocol (total of 30 h) in which they initially received the drink for 11 h instead of 38 h; the remainder of the protocol remained the same. Women continued to breastfeed their infants in a timed fashion from both breasts every 3 h throughout the duration of the study protocol, and were asked to empty their breasts of residual milk using a standard electric breast pump (Playtex,Dover, DE). While subjects were admitted to the GCRC, milk samples were collected every 3 h during the beginning, middle, and end of each infant feeding, and all samples from a given time point were pooled for analysis. Each subject received a breast shield and valve membrane that were initially sterilized by autoclaving for use in the duration of the study. Milk samples were collected into sterile conical tubes (15 mL) every 3 h throughout the duration of the study (approximately 72 h), and the samples collected at the completion of each dietary treatment were used for HMO and microbiome analysis. Human milk samples were then aliquoted into clean 4 mL plastic tubes. After completing the first dietary treatment, subjects were discharged from the GCRC and were told to resume their normal dietary intake and encouraged to keep their caloric dietary composition at 55% carbohydrate, 25% fat, and 15% protein. No measures of compliance to this request were made. Subjects were then readmitted 1–2 weeks later to receive the alternative treatment in the exact same manner, with the exception of using the other sugar. For each dietary treatment, the subjects were blinded to the sugar content (glucose versus galactose) of the drink.

*Carb/Fat Cohort* Details of this cohort and study design have been previously described^23^. Healthy, non-obese (BMI < 30) breastfeeding women were enrolled in this study (subjects H–N). Healthy was defined by: 1. normal physical examination, 2. normal results for liver function tests, fasting blood glucose, and hemoglobin concentrations. Additionally, all women had no history of gestational diabetes, nor family history of a first-degree relative with diabetes. Women were not taking any medications at the time of this study except for postpartum vitamin and mineral supplements, and all women had a negative pregnancy test. All women were between 6 and 14 weeks postpartum at the time of the study. Subjects were not selected on the basis of ethnicity, and included White, Non-Hispanic (n = 2), White, Hispanic (n = 3), and African American (n = 2) women. In the high fat dietary intervention, 1 subject’s sample (“subject N”) had an insufficient volume (< 20 μl) to rerun on HPLC for HMO speciation a when QC for a high-quality chromatogram failed^35^. As a result, subject N’s samples were excluded from HMO paired analysis but retained for the metagenomics where there was sufficient quantity and quality of sample.

Each subject was studied for 8 days on 2 separate occasions separated by a 1–2 week washout period. Subjects were randomly assigned to receive either a carbohydrate (60% carbohydrate, 25% fat, and 15% protein) diet or high fat (30% carbohydrate, 55% fat, and 15% protein) diet, followed by the alternative diet on the second occasion. The carbohydrate and high fat diets were isocaloric and isonitrogenous for each subject. Caloric content for each subject was set at 1.3 (low activity level) times the subject’s basal metabolic rate (BMR), calculated using the Harris Benedict equation:

$${\text{BMR}}\,\left( {\text{kcal/d}} \right) = 9.56 \times {\text{weight}}\,\left( {{\text{kg}}} \right) + 6.25 \times {\text{height}}\,\left( {{\text{cm}}} \right){-}4.68 \times {\text{age}}\,\left( {{\text{y}} + 655} \right).$$

No additional calories were provided to account for the caloric expenditure of lactation. After interviewing with a research dietitian, diets for each subject were created with the appropriate caloric content based on known macronutrient compositions of foods and subject preferences. Meals (3 per day) and snacks (3 per day) were weighed, prepacked and provided to the subject, and any unconsumed food was returned to the research kitchen. Subjects were not told which dietary treatment (high fat versus carbohydrate) they were consuming; however they may have been able to infer the dietary treatment from the food content. Subjects consumed the prepacked meals for 4 days at home and then were admitted to the GCRC. While at home, subjects were instructed to report if they consumed any additional food, though no independent assessment of their compliance to this instruction was made. After admission to the GCRC, subjects consumed the same meals as they did at home for an additional 3 days, feeding their infants every 3 h. Women then fasted overnight (13 h), and the next day consumed small meals every 15 min for 7 h which provided the same composition and caloric content as the meals provided the previous week; thus each woman consumed each diet for a total of 8 days. During the last 12 h of the study, isotopic infusions of glucose were performed for the kinetics studies previously described^23^. While subjects were admitted to the GCRC, milk samples were collected every 3 h (days 5–7) during the beginning, middle, and end of infant feeding, and all samples from a given time point were pooled for analysis. On day 8, milk samples were collected at 03:00, 09:00, 12:00, and 16:00. After the completion of the protocol, subjects were discharged home and were told to resume their normal dietary intake. They were encouraged to keep their caloric dietary composition at 55% carbohydrate, 25% fat, and 15% protein; however no measures of compliance to this request were made. After this 1–2 week washout period, subjects returned to receive the alternative diet following an identical protocol.

### HMO quantification

High-performance liquid chromatography (HPLC) was used to characterize HMO in human milk as previously described^35^ and when sufficient volume (20 μL) was available to obtain high quality chromatograms. HMO concentrations in paired milk samples from each subject in both the Glu/Gal Cohort and Carb/Fat Cohort were quantified using HPLC (Fig. 2a, Supplementary Table S2). In the high fat dietary intervention, 1 subject’s sample (“subject N”) had an insufficient volume (< 20 μl) to rerun a when QC for a high quality chromatogram failed^35^. As a result, subject N’s samples were excluded from HMO paired analysis but retained for the metagenomics where there was sufficient quantity and quality. Human milk (20 μL) was spiked with raffinose (a non-HMO carbohydrate) as an internal standard at the beginning of sample preparation to correct for sample losses during sample processing and allow for absolute oligosaccharide quantification. Oligosaccharides were extracted by high-throughput solid phase extraction over C18 (Hypercarb-96, 25 mg bed weight, thermo scientific) and Carbograph microcolumns (Hypersep-96 C18, 25 mg bed weight, thermos scientific) using a controlled vacuum manifold. Use of high-throughput microcolumns was validated in multiple different ways: (1) establishing parallelism in serial dilutions, (2) spiking milk with individual HMO standards to determine recovery, and (3) comparison with direct in-sample derivatization as used by others^59^. Oligosaccharides were fluorescently labeled with 2-aminobenzamide (2AB, Sigma) in a 96-well thermocycler at 65 °C for exactly 2 h. The reaction was stopped abruptly by reducing the thermocycler temperature to 4 °C. The amount of 2AB was titrated to be in excess to account for the high and variable amount of lactose and other glycans in milk samples. Unreacted 2AB was removed by high-throughput solid phase extraction over silica microcolumns (Hypersep silica, 25 mg bed weight, Thermo Scientific). Labeled oligosaccharides were analyzed by HPLC (Dionex Ultimate 3000, Dionex, now Thermo) on an amide-80 column (15 cm length, 2 mm inner diameter, 3 μm particle size; Tosoh Bioscience)) with a 50-mmol/L ammonium formate–acetonitrile buffer system. Separation was performed at 25 °C and monitored with a fluorescence detector at 360 nm excitation and 425 nm emission. Peak annotation was based on standard retention times of commercially available HMO standards (see Supplementary Table S6 for standard source and purity) and a synthetic HMO library^60^ and offline mass spectrometric analysis on a Thermo LCQ Duo Ion trap mass spectrometer equipped with a Nano-ESI-source. Absolute concentrations were calculated based on HMO standard response curves for each of the annotated HMO (standard response curves are shown in Supplementary Figure S7) and corrected for internal standard recovery. (Oligosaccharide detection limit: ~ 20 pmol, dynamic range between 20 and 5000 pmol; milk samples were diluted accordingly). The total concentration of HMO was calculated as the sum of the annotated oligosaccharides. The proportion of each HMO making up the total HMO concentration was also calculated. HMO-bound fucose was calculated on a molar basis. One mole HMO with one fucose residue counted as one mole HMO-bound fucose while one mole HMO with two or more fucose residues counted as two or more moles of HMO-bound fucose. The same was calculated for HMO-bound sialic acid.

### Secretor status determination

Maternal Secretor status was determined by the high abundance (Secretor) or near absence (Non-Secretor) of the HMO 2′-fucosyllactose in the respective milk samples with a cutoff of 100 nmol 2′FL/mL.

### Whole genome shotgun (WGS) microbiome analysis

DNA extractions were performed using the MoBio PowerSoil DNA Isolation Kit (MO BIO Laboratories). For each extraction, 300–400 μL of milk was extracted according to kit instructions with one exception: to improve DNA yield, the entire volume of the supernatant was added to a proportionate volume of C4 solution and then passed through the Spin Filter for typically 5 spins. In some cases, DNA from multiple extractions of the same sample was combined to achieve sufficient DNA quantity for sequencing. Kit negatives were included in each set of extractions (Glu/Gal: n = 4; Carb/Fat: n = 2) to evaluate potential bacterial contamination from the kit; 1 randomly selected of these kit negatives from each of the cohorts (total, n = 2) were subjected to parallel whole genome library builds and run on the same sequencing run as their designed subject samples (KitNeg1 and KitNeg 2 from supplementary Fig. 4). Minimal kit contamination was observed as determined by PCR amplification of the V1–V3 region of the 16S rRNA gene from samples and kit negative controls (Supplementary Fig. 4A). Primers amplifying the V1–V3 region of the 16S rRNA gene (27F-AGAGTTTGATCMTGGCTCAG and 534R-ATTACCGCGGCTGCTGG, expected band size 507 bp) were used with GoTaq DNA Polymerase (Promega) and the following reaction conditions: 95 °C for 10 min (1 cycle); 95 °C for 15 s, 60 °C for 1 min, 72 °C for 1 min (30 cycles); 72 °C for 5 min (1 cycle). PCR products were also assessed using a 2100 Bioanalyzer chip (Agilent Technologies) to determine precise amplicon size and to quantify band differences compared to 5 kit negative controls. No bands were detected by the automated band analysis (2100 Expert Software v.B.02.10.SI764, Agilent Technologies), nor were visible upon manual inspection of chromatograms (Supplementary Fig. 4B). Another primer set against the 16S rRNA gene was then used in TaqMan qPCR with the primers F-TCCTACGGGAGGCAGCAGT, R-GGACTACCAGGGTATCTAATCCTGTT, and probe (6-FAM)-5-CGTATTACCGCGGCTGCTGGCAC-3-(TAMRA) to most sensitively assess kit negatives against the human milk extractions for contaminating kit DNA. Probes were run according to standard Agilent StepOnePlus qPCR protocol at 60 °C for 40 cycles. For kit negatives, 2 of 5 extractions were undetected, while the cycle threshold for the remaining 3 was 8 cycles greater than the mean for human milk extractions (signifying approximately 250-fold less 16S rRNA signal compared to human milk samples) (Supplementary Fig. 4C).

WGS sequencing was performed at the Human Genome Sequencing Center (HGSC) at Baylor College of Medicine in the HiSeq 2000 platform (lllumina) for the Glu/Gal Cohort and HiSeq 2500 platform (Illumina) for the Carb/Fat Cohort; both were sequenced at a depth of 2 Gb per sample. For the Carb/Fat Cohort, an additional 1 to 4 cycles (above the standard 10 cycles) were added during library prep for several samples to achieve sufficient quantity for sequencing. Quality filtering and removal of human reads were performed using Kneaddata v0.5.2 (http://huttenhower.sph.harvard.edu/kneaddata) using BMTagger for human read removal and the following trimmomatic parameters: MINLEN 85 bp; SLIDINGWINDOW 4:20, which remove reads shorter than 85 bp and low quality reads, respectively. PhiX viral sequences from Illumina HiSEQ quality control as well as partial adapter sequences were identified and removed with BBDuk as implemented in BBTools (http://jgi.doe.gov/data-and-tools/bbtools/). Total read numbers before and after filtering are provided in Supplementary Table 4.

*Taxonomic classification* Kraken^61^ was used to assign taxonomy to filtered paired reads by mapping against the human and bacterial Kraken databases, and Bracken was then used to determine abundance of taxa by inferring corrected read counts for each taxa. To assess for the likely or potential contribution of kit contamination, we first compared the taxonomical content of the samples to the sequenced kit negatives (Supplementary Fig. 6A). Overall, the profile of the most abundant species in human milk did not correspond to the kit negatives, with the kit contaminants in greatest abundance being not represented in the human milk for the most abundant taxa. To control for the contribution of these taxa in silico decontamination was undertaken using the decontam R package (v0.99.1)^62^. Decontam makes statistical estimations of likely contaminants based on the relative abundances of shared taxa between kit controls and experimental samples (Supplementary Fig. 6B). For isNotContaminant the default parameters were employed (method = "prevalence", threshold = 0.5, normalize = TRUE, detailed = TRUE). Taxa identified as contaminants based on this software were removed from the taxonomic analysis (replotted in Fig. 3) Corrected read counts were then normalized to the total sum of non-human (bacterial) corrected read counts in each sample to determine relative abundance of each taxa.

*Metabolic pathway abundance and marker gene analysis* Concatenated paired reads filtered of human, PhiX, and partial adapter sequences were used as the input for metabolic pathway abundance using HUMAnN2^63^. To quantify the abundance of fucosidase and sialidase genes, ShortBRED was employed^42^. Specifically, ShortBRED markers for each gene family were generated using all of the bacterial proteins in the UniProt database classified under K01206 (fucosidase) and K01186 (sialidase), which were then mapped against the UniRef90 database to create a unique set of markers for each gene family^44^. Sequences that matched fucosidase and sialidase markers at 90% identity were then quantified in the metagenomic data set from each sample. To control for the varying sequencing depth across samples, ShortBRED hits for each protein family were divided by the total number of mapped bacterial reads in each sample to determine relative abundance of fucosidase or sialidase. Given the previous demonstration that individual KOs are poorly quantified in WGS metagenomic datasets with read depths less than 15,000 annotated reads^43^, we only included samples with > 15,000 mapped bacterial WGS reads in the ShortBRED analysis (Supplementary Table 4, Supplementary Fig. 5).

### Inferred metagenomics (used only with 16S data)

Given the high level of human DNA and relatively low bacterial biomass in human milk, it is difficult to obtain a high level of WGS sequencing coverage of human milk bacteria. We were unable to achieve the sequencing coverage necessary (> 15,000 mapped bacterial) reads for quantification of individual KO abundances by WGS for all samples (Supplementary Fig. 5). To allow for quantification of fucosidase in samples with fewer than 15,000 mapped bacterial reads, inferred metagenomics from 16S rRNA gene sequencing was additionally employed to quantify abundance of fucosidase. DNA extraction was performed as described above for WGS sequencing. For the Glu/Gal Cohort, different human milk aliquots from the same time point were used for WGS and 16S rRNA gene sequencing. For the Carb/Fat Cohort, due to sample availability, milk samples from different time points (separated by 4 h, as described in the Study Design section) were used for WGS and 16S rRNA sequencing. For both cohorts, the V4 variable region of the 16S rRNA gene was amplified by PCR using primers 515F (5′-GTGCCAGCMGCCGCGGTAA) and 806R (5′-GGACTACHVGGGTWTCTAAT) and sequenced using the 2 × 250 bp paired-end protocol in the MiSeq platform (Illumina). All subsequent analysis was performed using QIIME (v1.9.1). Merged reads were demultiplexed and quality filtered (phred quality score > 19). Total filtered and mapped reads are provided in Supplementary Table 4. Closed-reference OTU picking^64^ was performed with taxonomic classification by the GreenGenes Database version 13-5^65^ filtered at 97% identity. PICRUSt^43^ was then used to perform inferred metagenomics by calculating KEGG (Kyoto Encyclopaedia of Genes and Genomes) Orthology gene abundances, including calculation of fucosidase (K01206) inferred relative abundance.

### In vitro bacterial growth

The following strains used in this study were acquired from the American Type Culture Collection (ATCC): *Streptococcus thermophilus* ATTC 491, *Streptococcus mitis* ATCC 6249, *Streptococcus oralis* ATCC 35037, *Streptococcus parasanguinis* ATCC 15912, and *Streptococcus cristatus* ATCC 51100. *Staphylococcus epidermidis* and *Staphylococcus aureus* isolates were used (Versalovic lab, Texas Childrens Hospital and Baylor College of Medicine. Cultures were maintained by plating strains on Brain–Heart-Infusion (BHI) agar (Difco) and isolating single colonies. Colonies were grown in Thioglycollate medium (Sigma Aldrich, Cat. No. T9032) aerobically at 37 °C overnight, then all strains were adjusted to optical density (OD_600 nm_) 0.1 and sub-cultured into a fully defined media^65^. All the components of the synthetic medium were purchased from Sigma Aldrich. Lacto-*N*-fucopentaose I (> 95%, Carbosynth, Cat. No. OL05676) and 2′-fucosyllactose (> 95%,Carbosynth, Cat. No. OF06739) were individually added to the media each at a concentration of 3 mg/mL. Cultures were incubated in 96-well plates (Sigma) for 24 h aerobically at 37 °C on a Synergy H1 Hybrid Multi-Mode Microplate Reader (Biotek), and OD 600 nm and pathlength corrected (977/900) OD were recorded every 15 min. Carrying capacity was calculated from the corrected OD values for each well using the R package GrowthCurver v0.2.1^66^, with the default method for background correction (minimum value).

### Statistics

The majority of comparisons and graphical representations were carried out using R using packages as specified^67^. T-tests were performed in Graphpad Prism or Microsoft Excel and Mann–Whitney in R. For individual HMO, false discovery rate (FDR) was calculated using the R package p.adjust, method = “fdr.” Statistical significance between groups (dissimilarly, Binary Jaccard, Supplementary Fig. 2) was calculated using Kruskal–Wallis in R. All linear regression analysis was performed in Graphpad Prism or R, with error margins indicating 95% confidence intervals. All alpha and beta diversity calculations were performed using phyloseq and vegan based on the corrected read counts determined by Bracken, subsampled to a depth of 75 percent of the minimum number of sample reads^39,68^. PERMANOVA using vegan’s adonis function was used to evaluate differences in beta diversity and Mann–Whitney was used to evaluate differences in alpha diversity (Shannon diversity). LEfSe feature selection^40^ with LDA score > 2.0 and alpha value of 0.05 for the factorial Kruskal–Wallis test was used to evaluate significantly different taxa and pathways in dietary groups. For all statistical analysis, significance was defined as *p* < 0.05. As noted in the text, all non-secretor subjects were excluded from the microbiome analysis reported in Figs. 3b–e, 4, 5 and 6. Additionally, HMO quantification of one sample in the Carb/Fat Cohort failed and thus was not included in analysis involving HMO data. All statistical tests were calculated as two-sided. For in vitro culture results, Grubb’s test was used to determine outlier carrying capacity values which were subsequently removed. Additionally, any growth curves yielding a “questionable fit” by the GrowthCurver package were eliminated from analysis. The Shapiro–Wilk test was then used to determine if carrying capacity data was normally distributed for each group; student’s t-test was used for experiments with normally distributed data (*S. cristatus*, *S. oralis*, *S. parasanguinis*, *S. thermophilus*, *S. epidermidis*, and *S. aureus*), and the Mann–Whitney test was used for experiments with data that was not normally distributed (*S. mitis*—2′FL and LNFP I experiments).

## Supplementary Information

Supplementary Information.

Supplementary Table S2.

Supplementary Table S3a.

Supplementary Table S3b.

## Data Availability

Filtered WGS microbiome sequencing data and raw 16S rRNA microbiome sequencing data for all samples has been deposited in the National Center for Biotechnology Information (NCBI) Sequence Read Archive (SRA) under SRA accession SRP116264 and BioProject accession PRJNA400259.
