# Supplementary material for: Maternal diet alters human milk oligosaccharide composition with implications for the milk metagenome
Source: Sci Rep. 2020 Dec 16;10:22092. doi: 10.1038/s41598-020-79022-6 (PMC7745035; doi:10.1038/s41598-020-79022-6)
Supplement: Supplementary file 1 — Supplementary Information. [file 41598_2020_79022_MOESM1_ESM.pdf]

Supplementary Information Title Page

Title: Maternal diet alters human breastmilk oligosaccharide composition with implications for the milk metagenome

Authors: Maxim D. Seferovic*,[1,7] Mahmoud Mohammad*,[2,7] Ryan M. Pace*,[1,7] Melinda Engevik*,[3,7] James Versalovic,[3] Lars Bode,[4] Morey Haymond,[2] Kjersti M. Aagaard[#1,5,6]

Affiliations:

[1]Department of Obstetrics and Gynecology, Division of Maternal-Fetal Medicine, Baylor College of Medicine and Texas Children's Hospital, Houston, TX, 77030, United States

[2]Department of Pediatrics, Children's Nutrition Research Center, Baylor College of Medicine, Houston, TX, 77030, United States

[3]Department of Pathology and Immunology, Baylor College of Medicine and Texas Children's Hospital, Houston, TX, 77030, United States

[4]Department of Pediatrics, Division of Neonatology and Division of Gastroenterology, Hepatology and Nutrition and Larsson-Rosenquist Foundation Mother-Milk-Infant Center of Research Excellence, University of California San Diego, La Jolla, CA, 92093, United States

[5]Department of Molecular and Human Genetics, Baylor College of Medicine, Houston, TX, 77030, United States

[6]Department of Molecular and Cell Biology, Baylor College of Medicine, Houston, TX, 77030, United States

*[7]These authors contributed equally

[#]**Corresponding Author & Lead Contact:** Further information and requests for resources and reagents should be directed to and will be fulfilled by the lead contact:
Kjersti Aagaard, MD PhD FACOG
Professor and Vice Chair
Baylor College of Medicine
Division of Maternal-Fetal Medicine
One Baylor Plaza, Jones 314
Houston, TX, 77030

Phone: 713 798-8467
Email: aagaardt@bcm.edu

The authors have declared that no conflict of interest exists.

Supplemental Information – Table of Contents

**Supplementary Figure S1**	Concentrations of individual fucosylated and sialylated HMOs
**Supplementary Figure S2**	Taxonomic composition of the milk microbiome in secretors and non-secretors.
**Supplementary Figure S3**	Correlations between gene abundance (fucosidase and sialidase) and HMO-bound fucose and sialic acid, respectively and taxonomic differences between cohorts.
**Supplementary Figure S4**	Evaluation of bacterial DNA in samples and kit negatives.
**Supplementary Figure S5**	Evaluation of sequencing depth on ShortBRED quantification of fucosidase abundance.
**Supplementary Figure S6**	Initial species-level profile of milk microbiome and controls by WGS sequencing
**Supplementary Figure S7**	Standard response curve for HMO quanfification
**Supplementary Figure S8**	Literature comparison of HMO concentrations
**Supplementary Figure S9**	Assessment of HMO fucose and sialic acid changes by week postpartum

**Supplementary Table S1**	Characteristics of subjects in dietary cohorts.
**Supplementary Table S2**	Relative percent and molar concentration of HMOs – see Table_S2.xlsx
**Supplementary Table S3**	Relative abundance of bacterial taxa in milk microbiome determined by WGS sequencing – see Table_S3.xlsx
**Supplementary Table S4**	Read counts for WGS and 16S-V4 rRNA gene sequencing.
**Supplementary Table S5**	Fucosidase (K01206) hits detected by ShortBRED in Glu/Gal Cohort.
**Supplementary Table S6**	HMO standards - sources and purities

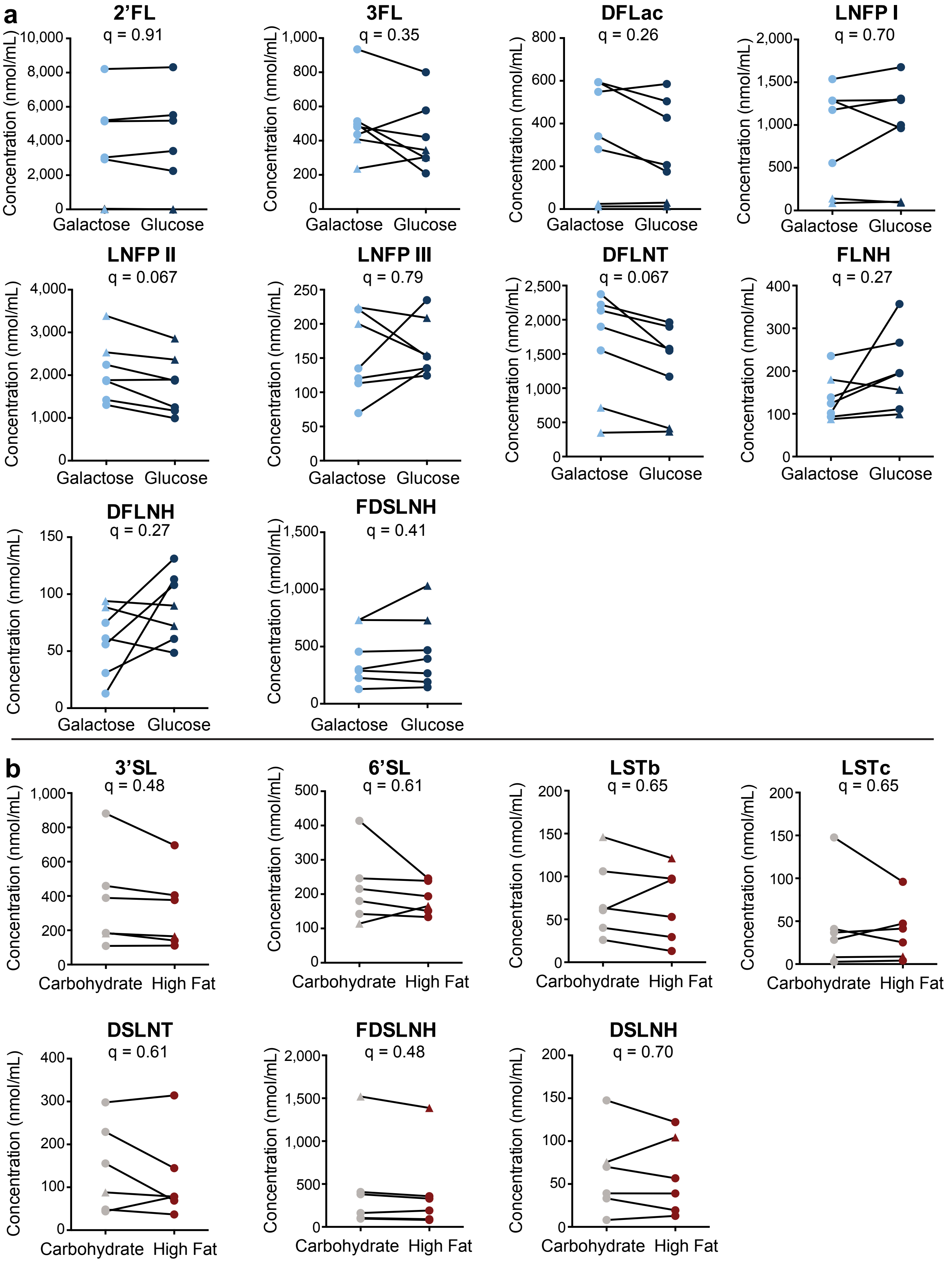

Supplementary Figure S1. Concentrations of individual fucosylated and sialylated HMOs. a, Concentrations of individual fucosylated HMOs in Glu/Gal Cohort. **b**, Concentrations of individual sialylated HMOs in Carb/Fat Cohort. Paired t-test followed by false discovery rate (FDR) correction were used to determine q values. Circles: Secretors; Triangles: Non-secretors. 2'FL: 2'-fucosyllactose, 3FL: 3-fucosyllactose, DFLac: difucosyllactose, LNFP: lacto-N-fucopentaose,DFLNT: difucosyllac-to-N-tetraose, FLNH: fucosyllacto-N-hexaose, DFLNH: difucosyllacto-N-hexaose, 3'SL: 3'-sialyllac-tose, 6'SL: 6'-sialyllactose, DSLNT: disialyllacto-N-tetraose, DSLNH: disialyllacto-N-hexaose, FDSLNH: fucosyl-disialyllacto-N-hexaose.

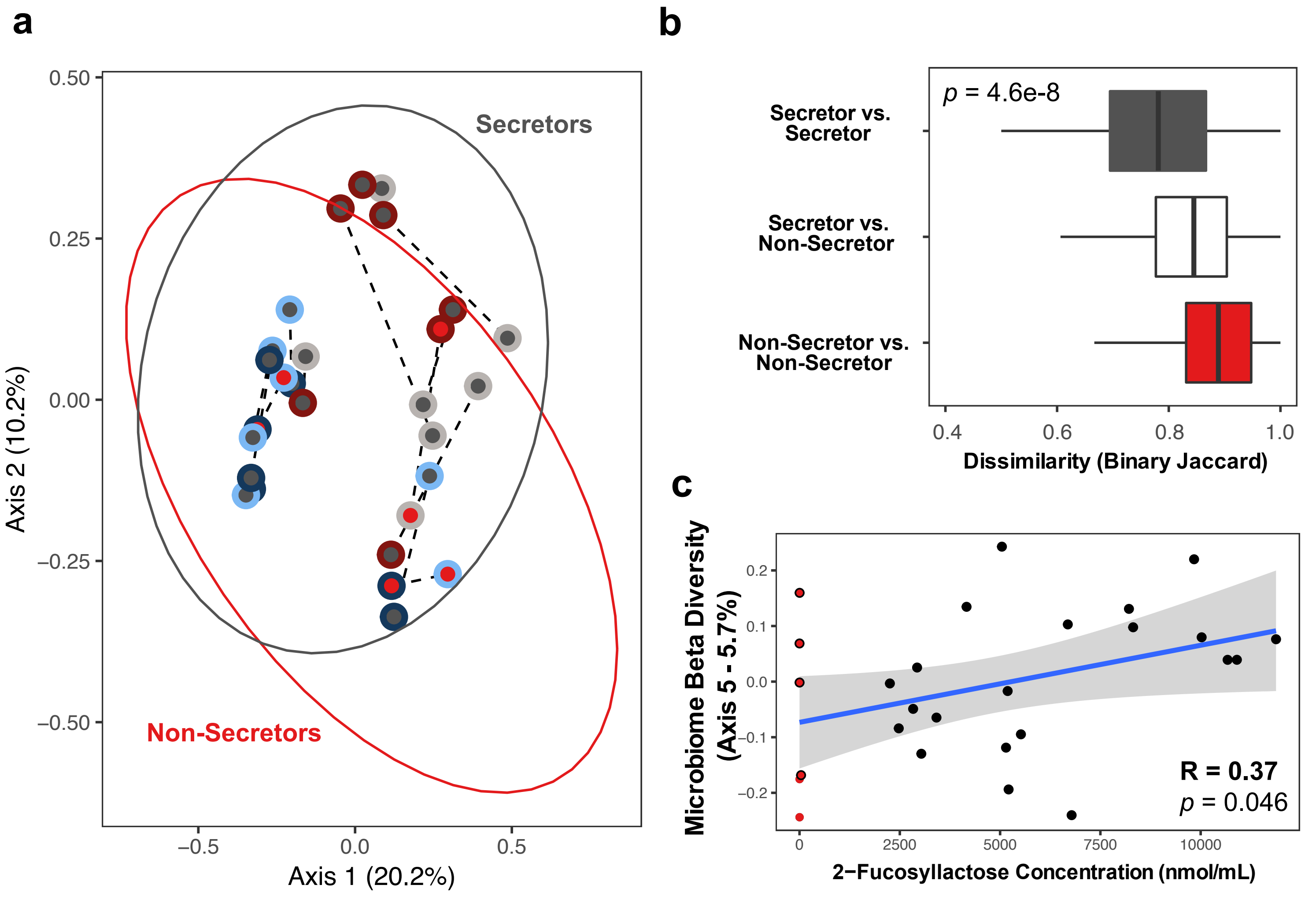

Supplementary Figure S2. Taxonomic composition of the milk microbiome in secretors and non-secretors. a, Principal coordinate analysis based on the presence or absence of each bacterial species (Binary Jaccard). Dotted connecting lines indicate paired dietary samples from the same subject, colored outlines indicate dietary treatment (dark red outline: high fat; grey outline: carbohydrate; dark blue outline: glucose; light blue outline: galactose; red fill: non-secretors, grey fill: secretors). Ellipses represent 955 confidence intervals. **b,** Pairwise comparisons of the dissimilarity of the milk microbiome of samples show that the milk microbiome of secretor samples is less similar to non-secretor samples compared to other secretor samples, and that the dissimilarity of the milk microbiome of non-secretor samples compared to other non-secretor samples is high, suggesting that non-secretors have a less consistent milk microbiome composition (p = 4.6e-8, Kruskal-Wallis test). **c,** Linear regression of the PC5 axis (MDS, Binary Jaccard) and 2'-FL concentration reveals an associa-tion between the taxonomic composition of the milk microbiome and 2'-FL concentration (linear regression, R = 0.37, p = 0.046), suggesting that the low concentration of 2'-FL in non-secretors may be influencing the milk microbiome in these subjects (red: non-secretor; black: secretor). The grey shaded area indicates the 95-percentile confidence bands.

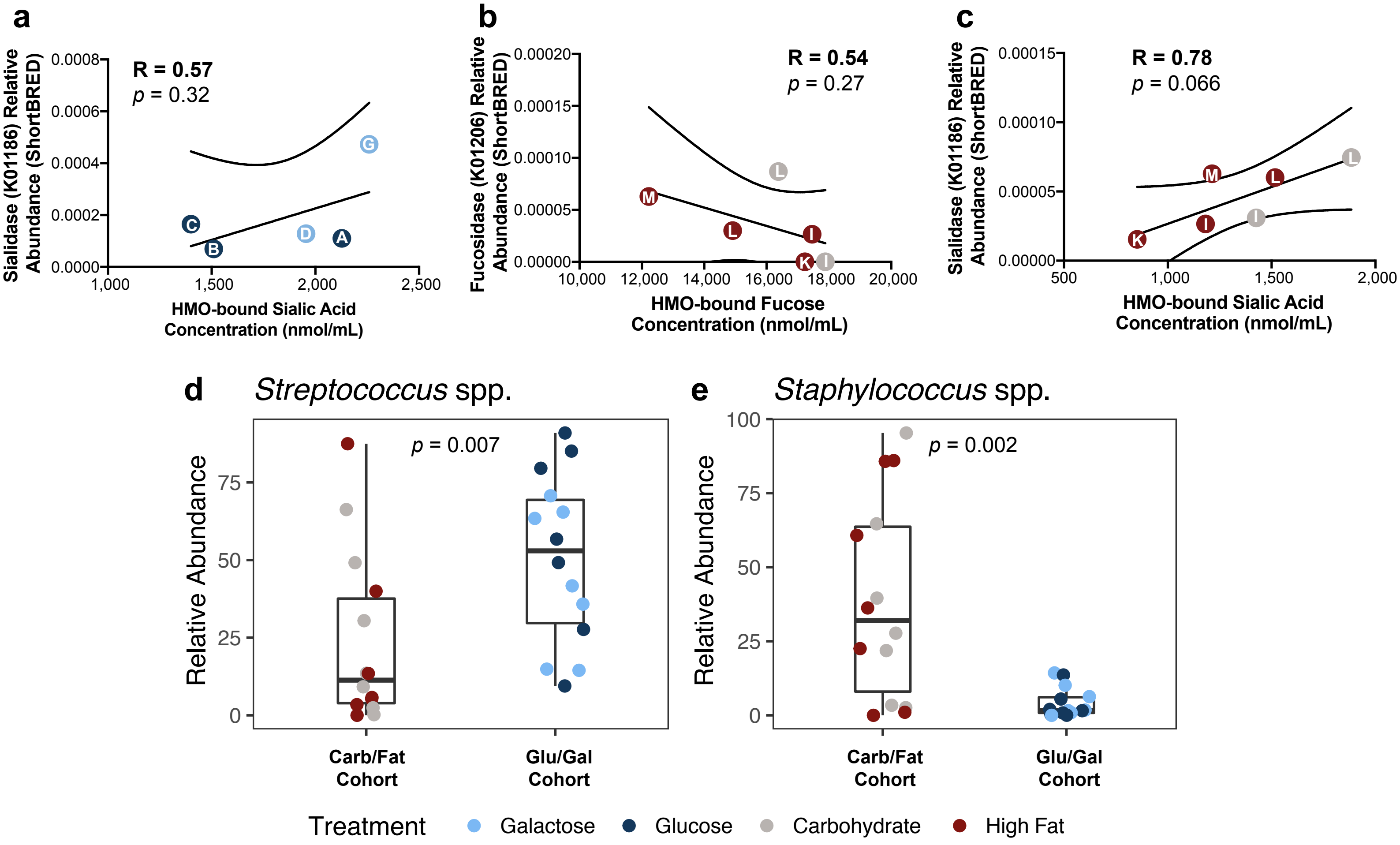

Supplementary Figure S3. Correlations between gene abundance (fucosidase and sialidase) and HMO-bound fucose and sialic acid, respectively and taxonomic differences between cohorts. a, No significant association detected between HMO-bound sialic acid and sialidase gene abundance in the Glu/Gal Cohort (linear regresssion, R = 0.57, *p* = 0.32). No significant association detected between fucosidase abundance and HMO-bound fucose concentration (**b**, linear regression, R = 0.54, *p* = 0.27) nor sialidase abundance and HMO-bound sialic acid concentration (**c**, linear regression, R = 0.78, *p* = 0.066) in the Carb/Fat Cohort. Cohorts significantly differ in taxonomic composition as revealed by the significantly higher levels of *Strepto-coccus* spp. in the Glu/Gal Cohort (**d**, Mann-Whitney test, *p* = 0.007) and significantly higher levels of *Staphylo-coccus* spp. in the Carb/Fat Cohort (**e**, Mann-Whitney test, *p* = 0.002). For panels a-c, gene abundances were determined by ShortBRED analysis and only samples with greater than 15,000 mapped bacterial WGS reads were included for analysis; letters indicate subject identifier. For all panels, colors indicate dietary treatment; dark blue: glucose diet, light blue: galactose diet, gray: carbohydrate diet, red: high fat diet. Error bars indicate in panels **a-c** indicate 95% confidence intervals; error bars in panels **d-e** indicate mean ± standard deviation.

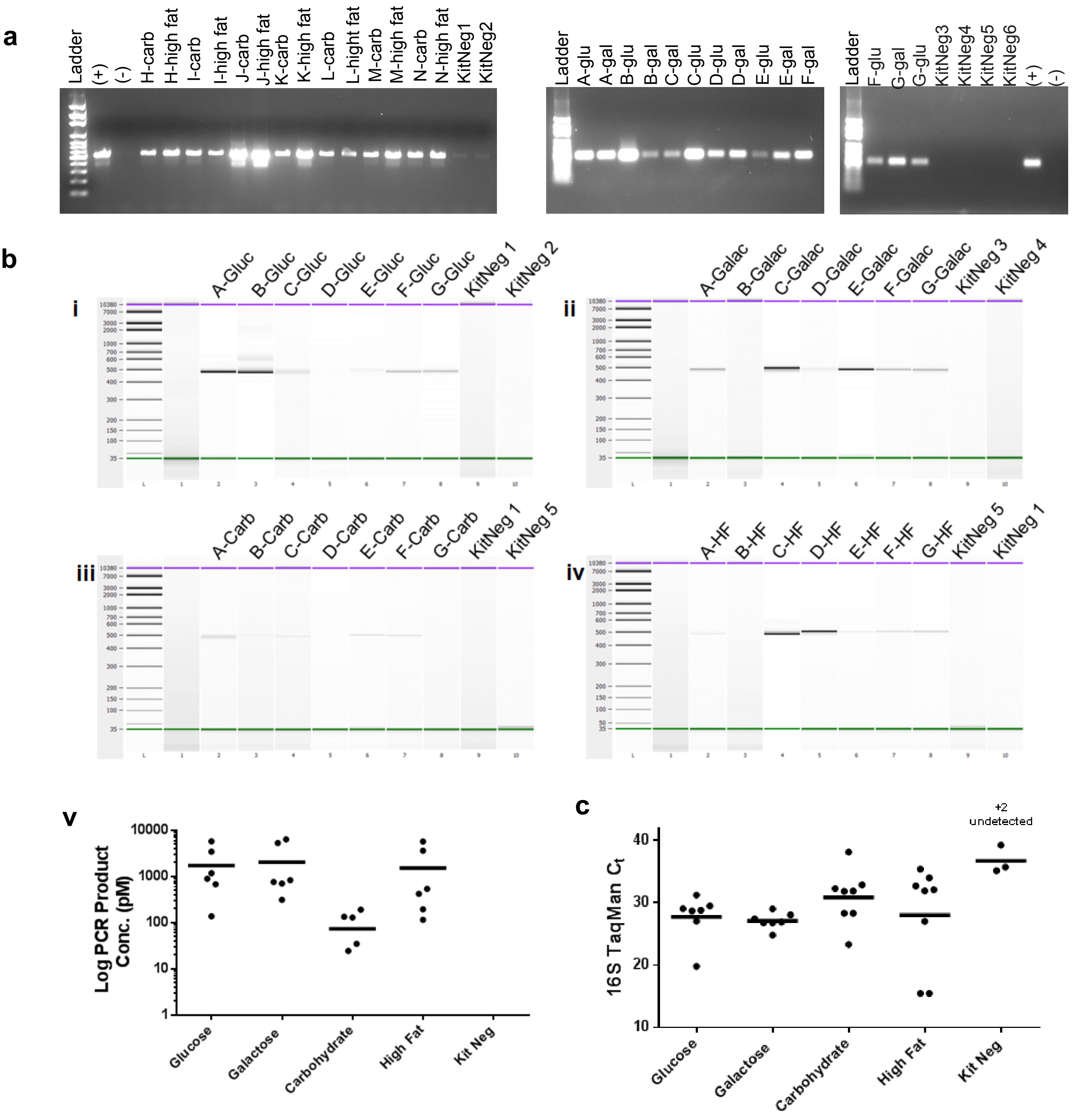

Supplementary Figure S4. Evaluation of bacterial DNA in samples and kit negatives. (A) PCR amplification of V1-V3 region of 16S rRNA gene in all samples (left: Carb/Fat Cohort; middle and right: Glu/Gal Cohort) and associated kit negative controls (no sample added, blank kit extraction). Labels indicate subject identifiers (A-N) and dietary treatment, kit negative controls, and PCR controls. **(B)** PCR products were assessed by Agilent ioanalyzer chip (i-iv). Qualification of the product band concentration was made (v). **(C)** A separate taquman qPCR against the 16S gene was done to quantifiy the relative bacterial signal in the breast milk samples.

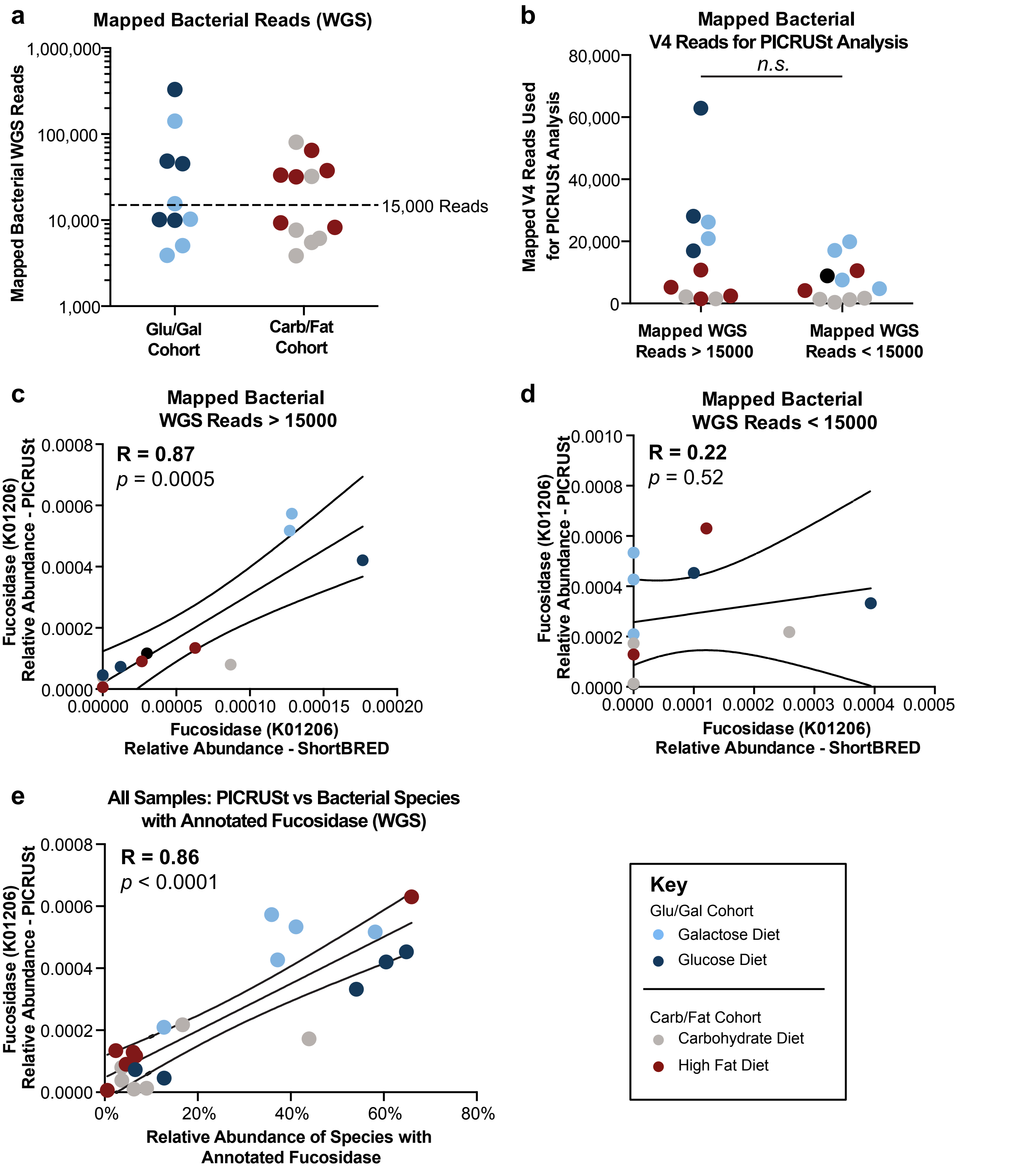

Supplementary Figure S5. Evaluation of the effect of sequencing depth on ShortBRED quantification of fucosidase abundance. a, Number of mapped bacterial WGS reads in each sample reveals several samples have fewer than 15,000 mapped bacterial reads, the number necessary to accurately quantify individual KO abundances in WGS metagenomic data. **b**, No significant difference in the number of 16S-V4 reads mapped to bacteria for samples with < 15,000 mapped bacterial WGS reads and samples with > 15,000 mapped bacterial WGS reads (Mann-Whitney test, $p = 0.17$). **c**, Strong association between ShortBRED and PICRUSt fucosidase abundance quantification in samples with > 15,000 mapped bacterial WGS reads (linear regression, R = 0.87, $p = 0.0005$) . **d**, Poor association between ShortBRED and PICRUSt fucosidase abundance quantification in samples with < 15,000 mapped bacterial WGS reads (linear regression, R = 0.22, $p = 0.52$). **e**, Tight correlation between PICRUSt quantifi-cation of fucosidase abundance and the relative abundance of bacterial species identified by WGS with fucosidase proteins in the UniPROT database (linear regression, R = 0.87, $p < 0.0001$). Error bars incidate 95% confidence intervals.

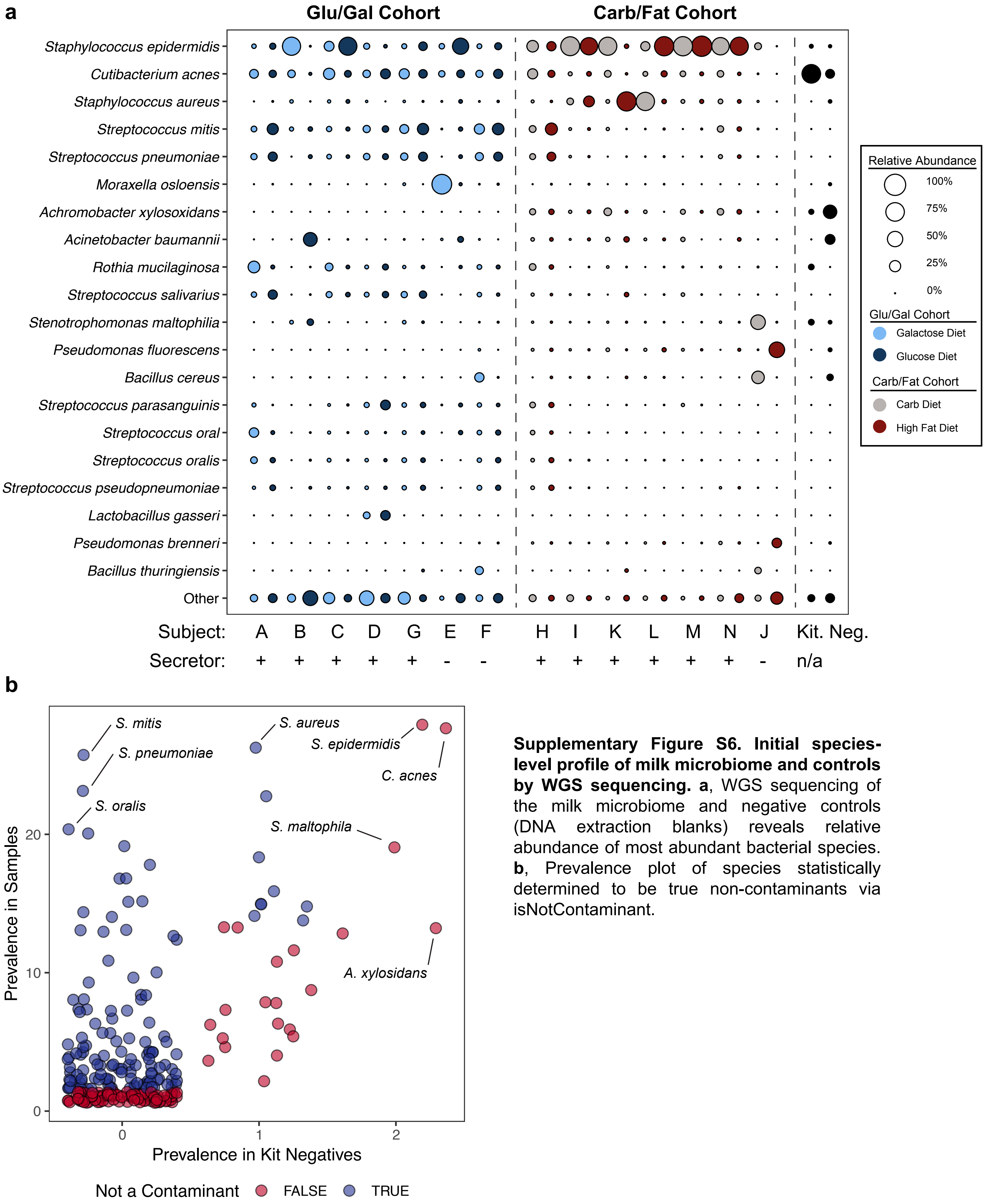

Supplementary Figure S6. Initial species-level profile of milk microbiome and controls by WGS sequencing. a, WGS sequencing of the milk microbiome and negative controls (DNA extraction blanks) reveals relative abundance of most abundant bacterial species. **b**, Prevalence plot of species statistically determined to be true non-contaminants via isNotContaminant.

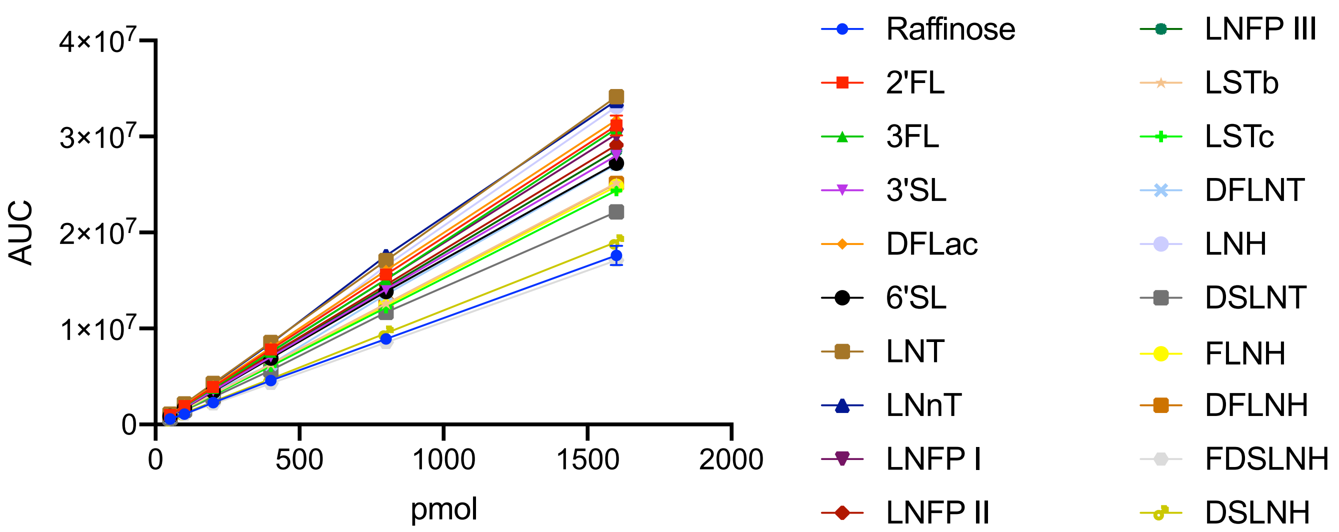

Supplementary Figure S7. Standard response curve for HMO detection. Area under the curve is measured for known concentrations of standards for 20 HMO species. Absolute concentrations were calculated based on HMO standard response curves for each of the annotated HMOs shown. The oligosaccharide detection limit was determined to be approximately 20 pmol, with a dynamic range between 20 and 5,000 pmol.

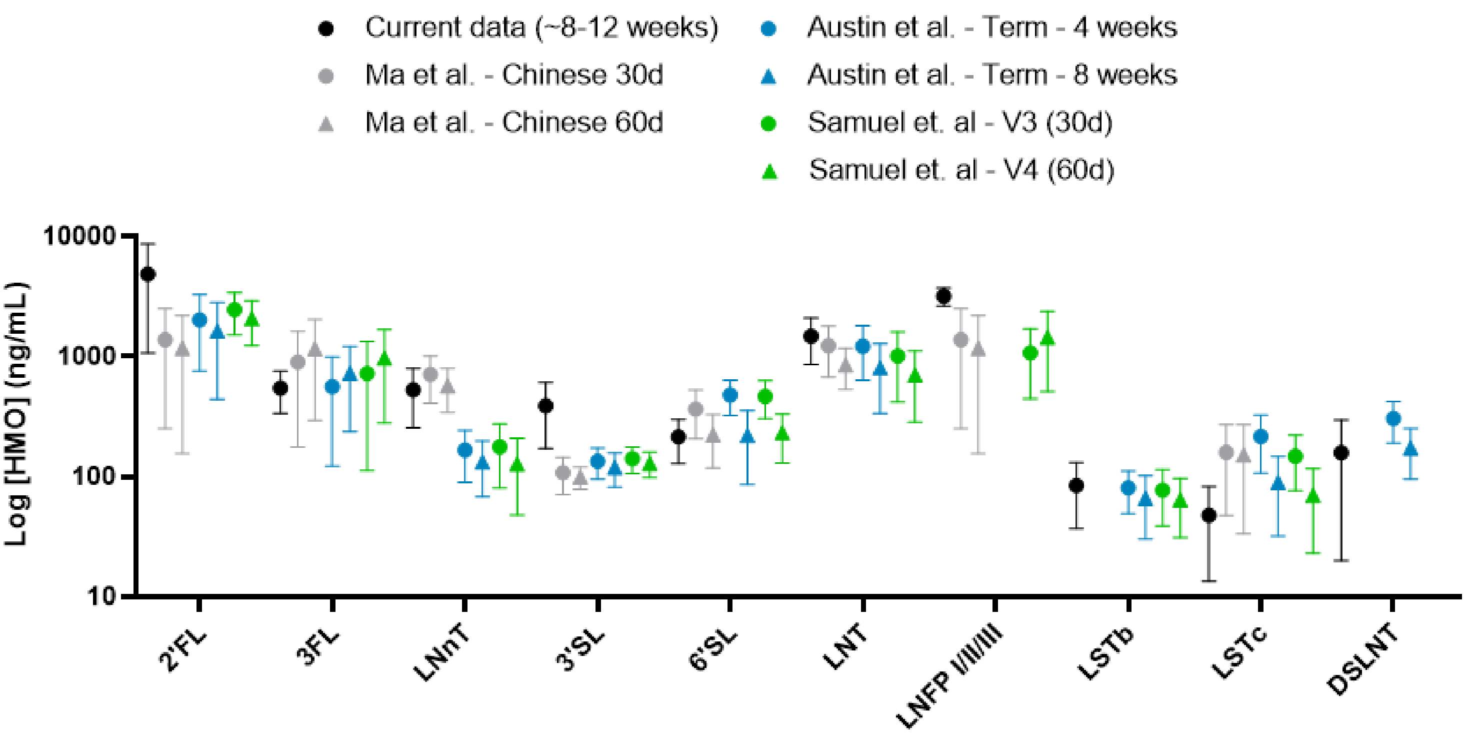

Supplementary Figure S8. Literature comparison of absolute HMO concentrations in the current study. The levels of HMO were compared for multiple time points to three other prominent, well described studies that quantified HMO in absolute terms. Raw data was taken from the supplemental materials of the indicated publications. Data that was selected from collected from breast milk at approximately 30 and 60 days. From Ma et al. Chinese data but not Malaysian was used as it contained both relevant time points. For Austin et al. reported data from preterm pregnancies was excluded. For Samuel et al. reference values across 100s of samples and multiple european countries is summarized.

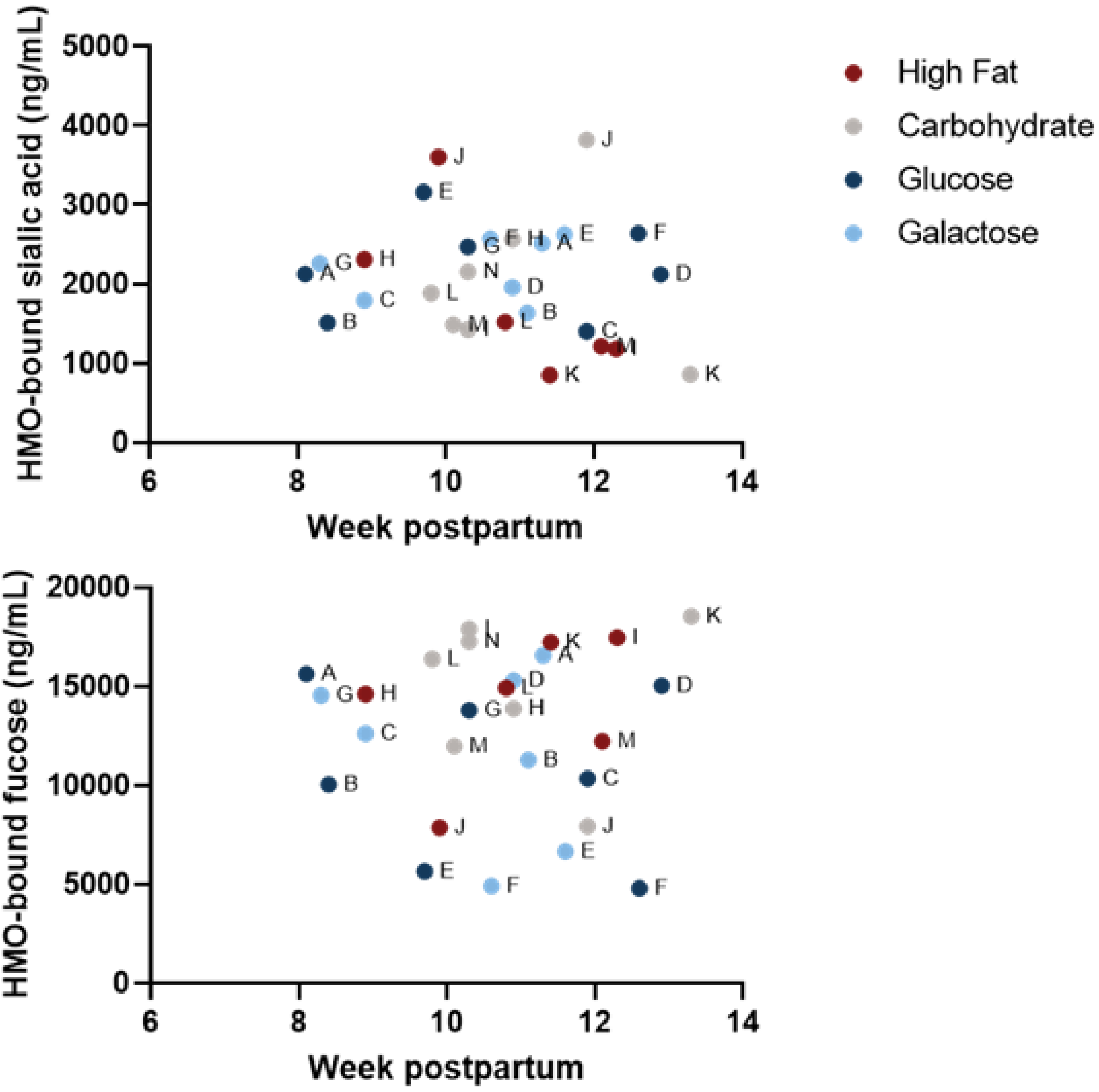

Supplementary Figure S9. Assessment of HMO fucose and sialic acid changes by week postpartum. The concentration of sialylated and fucosylated HMO is plotted by sample collection date for both diet groups. To control for the possible effects of changing HMO with week postpartum, within diet groups, half of women were randomized to receive one or the other diet at each visit. Spearman's correlation was performed to determine a relationship to week postpartum. No significant correlation was found when assessed either by diets individually or isolated by diet cohort, or all combined (r =-0.19, p=0.33, r=0.07, p=0.74 for Sialic acid and fucose bound respectively) for either HMO bound saccharide.

Supplementary Table S1. **Characteristics of subjects in dietary cohorts.**

Characteristic	Glu/Gal Cohort (*n* = 7)	Carb/Fat Cohort (*n* = 7)	Significance of Difference (p)
**Maternal Age (years)**	26.6 ± 3.5	29.3 ± 2.6	0.13[a]
**BMI (kg/m^2)**	34.5 ± 4.6	23.2 ± 1.7	0.0003[a]
**Weeks postpartum**	9.3 ± 1.4	10.1 ± 0.9	0.20[a]
**Secretor Status**	5 Secretor<br>2 Non-Secretor	6 Secretor<br>1 Non-Secretor	0.51[b]
**Ethnicity**	4 Hispanic<br>3 Non-Hispanic	3 Hispanic<br>4 Non-Hispanic	0.59[b]
**Race**	6 White<br>1 African American	5 White<br>2 African American	0.51[b]

[a]Determined by Student's t-test; [b]Determined by Chi-squared test. Ranges indicated mean ± standard deviation.

Supplementary Table S4. Read counts for WGS and 16S-V4 rRNA gene sequencing.

Sample	WGS Paired Raw Reads	WGS Filtered Paired Reads	WGS Mapped Bacterial Reads (Kraken)	Good's Coverage (%)	16S-V4 Quality Filtered Paired Reads	16S-V4 Mapped Reads
Subject A - galactose	13605054	36105	10257	100.00	9043	8910
Subject A - glucose	17910424	91883	45303	100.00	17176	16952
Subject B - galactose	17489673	27005	3902	99.99	4916	4787
Subject B - glucose	18785466	583959	329537	100.00	63957	62913
Subject C - galactose	19124015	28722	5047	100.00	7683	7538
Subject C - glucose	14724410	76996	48423	100.00	28231	28115
Subject D - galactose	14681864	117780	15558	99.97	21058	20902
Subject D - glucose	17624305	36132	10157	99.80	17265	17077
Subject E - galactose	15817538	45498	20734	99.95	35982	35877
Subject E - glucose	15359991	19037	2208	99.99	2666	2503
Subject F - galactose	17179422	62892	28678	100.00	29733	29520
Subject F - glucose	16369384	46364	17629	100.00	15900	15647
Subject G - galactose	16895988	304791	141629	99.95	26383	26241
Subject G - glucose	16793749	35402	9977	100.00	20103	19927
Subject H - carbohydrate	8291980	25399	7661	99.97	383	375
Subject H - high fat	8677786	22373	8265	100.00	4139	4137
Subject I - carbohydrate	41205688	95039	32341	99.99	1496	1494
Subject I - high fat	16069013	65378	37596	100.00	2497	2454
Subject J - carbohydrate	27420383	511949	436940	100.00	23577	23555
Subject J - high fat	13627756	12061058	7928775	100.00	276185	276003
Subject K - carbohydrate	7746674	16560	6164	100.00	1262	1216
Subject K - high fat	5699115	81805	64920	99.99	10807	10791
Subject L - carbohydrate	11638433	129691	80466	99.99	2185	2183
Subject L - high fat	11448878	62963	33330	99.99	5281	5244
Subject M - carbohydrate	6583151	14651	5503	99.98	1361	1347
Subject M - high fat	6013984	46103	31821	100.00	1521	1517
Subject N - carbohydrate	4114285	9883	3868	100.00	1700	1698
Subject N - high fat	6125003	22465	9316	99.94	10600	10589

Supplementary Table S5. **Fucosidase (K01206) hits detected by ShortBRED in Glu/Gal Cohort.**

Subject A - Glucose	Subject B - Glucose	Subject C - Glucose	Subject D - Galactose	Subject G - Galactose
8	4	0	2	18

Only samples with greater than 15,000 mapped bacterial WGS reads are included.

Supplementary Table S6. HMO standards - sources and purities

HMO	Source	Purity
2’FL	Sigma	>95% by NMR
3FL	Elicityl	>90% by NMR
DFLac	Elicityl	>95% by NMR
3’SL	Sigma	>97% by HPLC
6’SL	Sigma	>98% by HPLC
LNT	Elicityl	>95% by HPLC
LNnT	Elicityl	>95% by HPLC
LNFP1	Dextra	>95% by HPLC
LNFP2	Dextra	>93% by HPLC
LNFP3	Dextra	>90% by HPLC
DFLNT	Dextra	>90% by HPLC
LSTb	Dextra	>93% by HPLC
LSTc	Dextra	>90% by HPLC
DSLNT	Dextra	>93% by HPLC
LNH	Elicityl	>94% by HPLC
FLNH	Dextra	>90% by HPLC
DFLNH	Dextra	>90% by HPLC
DSLNH	Dextra	>94% by HPLC
FDSLNH	Purified	>95% by HPLC
